# 3rd generation MICA with the “K-wires-first technique” - a step-by-step instruction and preliminary results

**DOI:** 10.1186/s12891-021-04972-5

**Published:** 2022-01-18

**Authors:** Andreas Toepfer, Michael Strässle

**Affiliations:** Department of Orthopaedic Surgery and Traumatology, Kantonsspital St. Gallen, CH-9007 St. Gallen, Switzerland

**Keywords:** Bunion surgery, Hallux valgus surgery, MICA, Minimally-invasive foot surgery, Percutaneous foot surgery, Surgical technique

## Abstract

**Background:**

Minimally-invasive techniques for hallux valgus correction are becoming increasingly popular. In the last decades, multiple techniques for minimally-invasive hallux valgus correction have been described. MICA (Minimally-invasive Chevron & Akin), representing the 3rd generation of minimally-invasive hallux valgus correction, combines the advantages of an extraarticular osteotomy, stable internal fixation, and high potential for correction.

This report aims to provide a step-by-step instruction of the surgical technique with the “*K-wires-first*” MICA modification, illustrated by detailed imaging of both intraoperative fluoroscopy and clinical imaging as well as corresponding sawbone models for each step. Preliminary results including radiological outcome and complications of the first 50 cases will be discussed.

**Methods:**

Between May 2018 and May 2021, 50 consecutive MICAs in 47 patients were performed with the *K-wires-first technique*. There were 40 women and 7 men with an average of 57.4y (range 25–78). The mean preoperative IMA was 16.2° (range 11.0–21.5), the HVA 30.6° (range 21.8–42.1).

**Results:**

There was one intraoperative conversion to an open surgical bunion correction corresponding to a 2% conversion rate respectively (1/50). On 3 feet (2 patients), removal of the Chevron screws was performed after 7, 9, and 12 months due to prominent and disturbing screw heads at the level of the medial cortex, accounting for a revision rate of 6% (3/50). There were no other secondary revision surgeries. The IMA decreased after MICA by a mean of 10.8° from 16.2° to 5.4° and the HVA by a mean of 22.1° from 30.6° to 8.5°, demonstrating MICA’s high potential for correction.

**Conclusions:**

Compared to other MICA techniques, the *K-wires-first* modification helps to reduce hardware malpositioning and the risk of conversion to open surgery. Furthermore, our preliminary results demonstrate a high potential for correction even for severe hallux deformities.

**Trial registration:**

Retrospectively registered, swissethics BASEC-ID 2021–01537, July 16th, 2021 (www.raps.swissethics.ch).

**Supplementary Information:**

The online version contains supplementary material available at 10.1186/s12891-021-04972-5.

## Background

The first-generation percutaneous technique was described by Isham in the early 90ies and included mainly the Reverdin-Isham procedure for the treatment of hallux valgus [1]. The Reverdin-Isham procedure consists of an intra-articular medial closing wedge osteotomy of the distal metatarsal performed without fixation and aims to align the first ray by medial rotation of the first metatarsal head and distal metatarsal articular angle (DMAA) [2]. Nowadays, Boesch osteotomy and, amongst other modifications, the Endolog technique are considered as 2nd generation MIS hallux valgus surgeries [3]. Those procedures require a small open-surgical incision and a transverse osteotomy which is stabilized with a percutaneous K-wire (Boesch) or a curved titanium endomedullary nail device (EndoLog) [4, 5]. Redfern and Vernois first described a surgical procedure for percutaneous hallux valgus correction that offered an extracapsular osteotomy, stable internal fixation, and the potential for correction of severe deformities in 2015 which is now considered a 3rd generation procedure [6–8]. Their MICA technique involves fixation of the Chevron osteotomy with two parallel screws, the use of another screw for the Akin osteotomy, and length preservation of the first ray [3, 9]. Ever since, several modifications of the original technique have been described, often deviating from the inaugurator‘s principles [10–13]. Failure to differentiate between various modifications and even different generations of MIS hallux valgus surgery in recent reviews led to inconclusive and sometimes confusing results, often comparing apples to oranges [14, 15].

This report focuses on a modified 3rd generation MICA procedure (*K-wires-first* technique). Key elements of this modification include a patient positioning that allows for easy intraoperative radiographic control with a standard-sized C-arm (without moving the fluoroscope) and a different sequence of surgical steps. The osteotomy is only performed once the K-wires are positioned perfectly, which are later replaced for the guidewires of the cannulated screws. This process aims to diminish hardware malpositioning and conversion to open surgery. Pearls and pitfalls as well as advantages and disadvantages of our modification are presented in the supplementary material section as tables (Tables 1 and 2). In the original technique as described by Redfern & Vernois, the osteotomy is performed first and the guide wires for the cannulated screws are introduced afterwards [7, 16].

At this point, it must be stressed that MICA does not represent a beginner’s procedure and is generally regarded as one of the more advanced and demanding percutaneous surgery techniques [17, 18]. MICA should not be embraced without adequate knowledge and training. This can be achieved by visiting dedicated MIS courses where the technique can be trained with the help of sawbone and cadaveric specimen and under the guidance of experienced surgeons [19]. Furthermore, fellowship training or visitation at a clinic that performs this procedure regularly can greatly improve the understanding of the pearls and pitfalls associated with live surgery.

## Methods

This study was a prospective observational single-surgeon case series of consecutive patients. Between May 2018 and May 2021, 50 consecutive MICAs in 47 patients (3 bilateral, 24 right, 20 left feet) were performed by the author with the *K-wires-first* technique. There were 40 women and 7 men with an average age of 57.4y (range 25–78). For demographics, please see Table 3 in the supplementary material section. In 5 out of 50 feet, no Akin osteotomy was necessary. All other cases included two screws for the Chevron and 1 screw for the Akin osteotomy.

Written informed consent from the patients and approval from the institutional review board were obtained. Key elements of the study include a short-term radiological outcome analysis and the assessment of surgery-related complications. Emphasis is placed on the evaluation of correct screw placement and the rate of revision surgeries, as our hypothesis states that the *K-wires-first technique* helps to reduce hardware malpositioning. Deformity correction was measured on weight-bearing preoperative and 6-week postoperative radiographs (without hallux tapes applied). For radiological evaluation, we used Mitchell’s method where the centre points of the proximal and distal articular surfaces of the first and the second metatarsal bone and the proximal phalanx are connected to form the lines [20]. As previously suggested, this method gives the most reliable postoperative angles as it relies on anatomical landmarks that are corrected along with the deformity [21–23]. The severity of the deformity was categorized according to AOFAS criteria into three grades [24]: mild (HVA < 20°, IMA 9°- 11°), moderate (HVA 20°- 40°, IMA 12°- 16°) and severe (HVA > 40°, IMA > 16°).

Parameters were expressed as mean, standard deviation (SD) and range (min./max.). Simple paired t-tests were used to determine the statistical significance of the difference between pre- and postoperative radiographic outcomes (IMA and HVA). A *p*-value < 0.05 was used to define statistical significance. Statistics were performed by an independent statistician using Microsoft Excel© and RStudio software.

Inclusion criteria consisted of any patient (min. age 18y) undergoing 3rd generation MICA and giving consent to participate. Exclusion criteria comprised of other techniques of hallux valgus surgery, including minimally-invasive Chevron osteotomies with only one cannulated screw and Akin osteotomies without screw fixation. Moreover, radiographical detection of severe osteoarthritis of the MTP1 joint and general contraindications for elective orthopedic foot surgery were considered contraindications for MICA. A history of prior surgery at the target site (e.g. open surgical hallux repair with hallux valgus recurrence) and concomitant fore- or hindfoot surgery (e.g. DMMO, MIS lesser toe correction, MICO) were no exclusion criteria. The use of consecutive prospectively collected data reduced selection bias [17].

### Indication for MICA

Indications and contraindications for MICA are discussed controversially in the sparse literature. We present our process of decision-making without claim to general validity. Indication for MICA was any hallux valgus deformity, independently from the grade of deformity (mild, moderate, severe) [24], with a stable TMT1 joint (see below). As demonstrated by Redfern & Vernois, MICA is suitable for severe deformities [6].

### Contraindication

A severe hypermobility / instability in the TMT1 joint, that can not be stabilized by activation of the peroneal muscle group is deemed to be unsuitable for MICA in our patients. Similar but less frequent, a hallux valgus with severe arthritic degeneration of the TMT1 joint requiring fusion is not suitable for MICA. These pathologies will be corrected with Lapidus fusion in our clinic. A severely pathological DMAA might be more difficult to fully correct percutaneously and, similar to hallux valgus recurrence after previously failed bunion surgery, is regarded as a relative contraindication. Nevertheless, with increasing experience, even these conditions can be successfully corrected by MICA. Degenerative changes inside the MTP1 joint can not be addressed as easily as in open surgery and might favor an open approach in selected cases.

## Surgical technique

### Preoperative planning

**Fig. 1:**
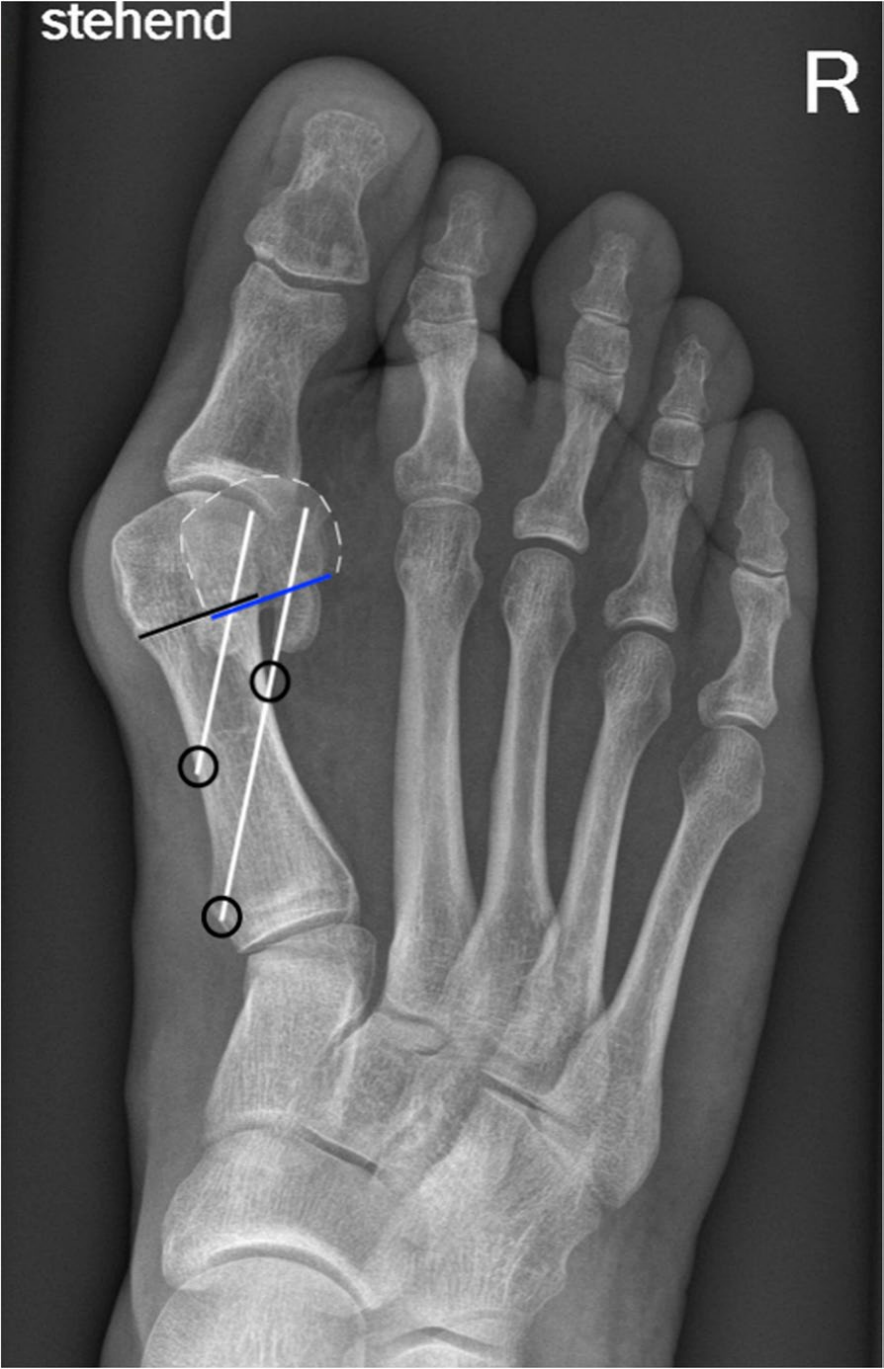
Preoperative planning of the Chevron osteotomy: The black line indicates the level and length of the osteotomy. The blue line has the same length as the black. The overlap of both lines (black&blue) simulates the amount of the lateral shift in relation to the width of the metatarsal shaft at the level of the osteotomy (in this case ~ 60–70%). The white lines simulate the orientation and length of both screws. The black circles demonstrate the cortical fixation points of the screws. The proximal screw exists through the lateral cortex, the distal screw through the osteotomy

Weight-bearing plain radiographs of the affected foot are obtained. The dorso-plantar x-ray is used simulating the correct placement of the screws, including estimated length, entry points, and angulation as well as the required extent of the shift of the 1st metatarsal head. The amount of the displacement of the metatarsal head is determined by the required coverage of the sesamoids. Contrary to an open Chevron osteotomy, a displacement of 80–100% or more (in relation to the width of the metatarsal shaft at the height of the osteotomy) is easily feasible with 3rd generation MICA procedures. Thus, the intermetatarsal angle I/II may be less relevant than the width of the 1st metatarsal bone and the first webpace. Although not required in each and every case, the Akin osteotomy is not only advised to correct a pathological hallux valgus interphalangeus but facilitates the centering of the (extrinsic) tendons that insert at the hallux and can act as a bowstring enhancing the development of a bunion recurrence. Furthermore, an Akin osteotomy can improve the aesthetical result. Thus, Akin osteotomy might still be indicated and advised despite a physiological hallux valgus interphalangeus angle. A strong and reliable three-point fixation (medial cortex, lateral cortex, 1st metatarsal head) provided by the proximal screw and additional two-point fixation of the second, distal anti-rotation screw (medial cortex and metatarsal head) is desired to enable a displacement of more than 50%. When planning and performing the surgery, it must be ensured that the proximal screw is best placed through both cortices and does not exit the metatarsal shaft through the osteotomy to guarantee maximum stability of the screw fixation (Fig. 1).

### Patient positioning & preoperative preparations

**Fig. 2:**
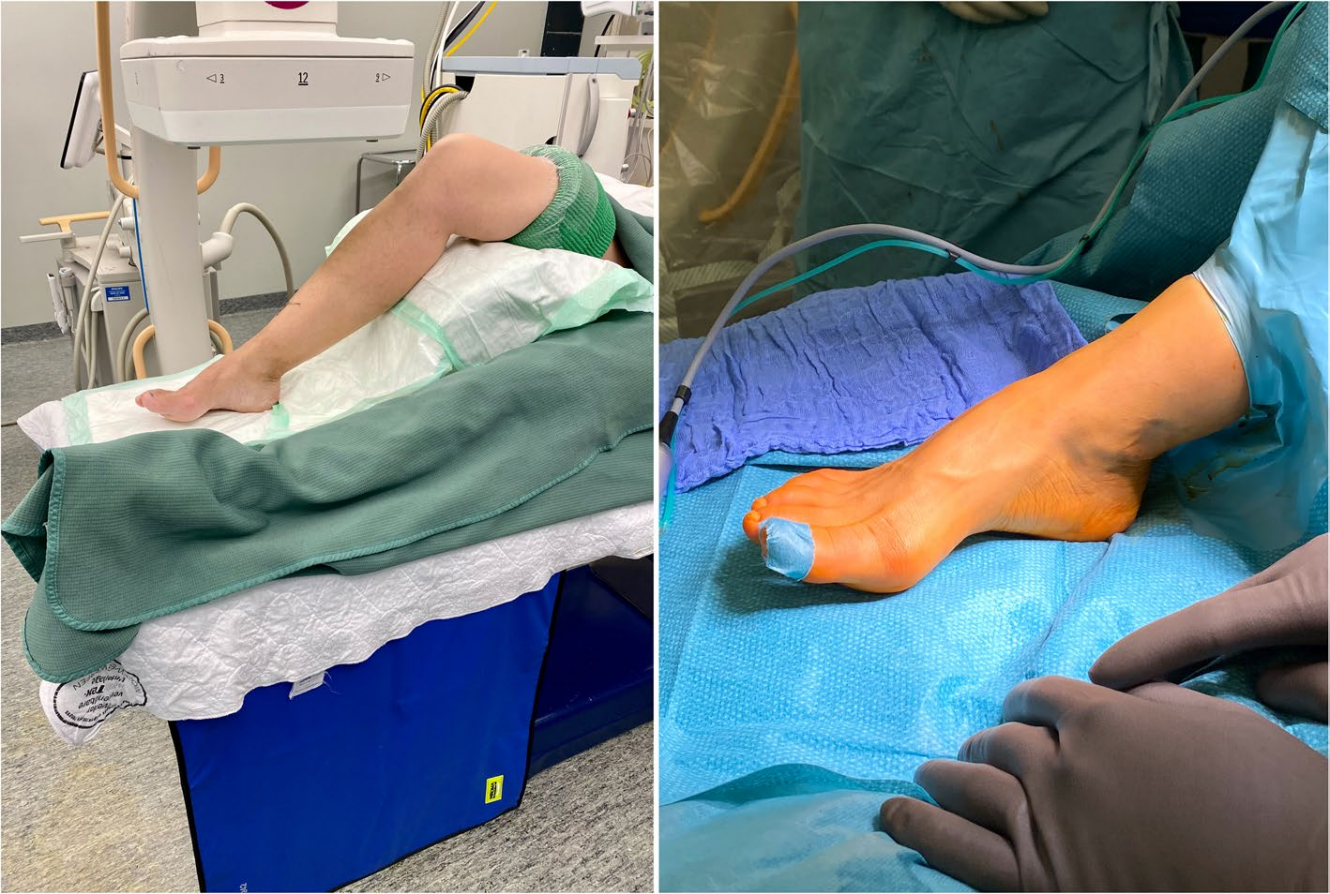
Modified patient positioning using a standard-sized C-arm before (left image) and after (right image) sterile draping: Hip and knee are flexed to allow the foot being placed in a plantigrade position on the operation table, parallel to the floor and detector of the fluroscope. Thereby easy manouverability around the foot is possible for adjunct procederes (e.g. DMMO) without moving the C-arm. By performing an external rotation in the hip a true lateral view is attainable. The oblique view is obtained by slight internal rotation in the hip joint. Note the *drape under table* radiation protection

After anaesthesia, which is performed routinely as an ischiofemoral nerve block in our clinic, the patient is placed in a supine position on a radiolucent table. With the help of an adequate support under the knee, the affected leg is flexed in the hip and knee so the foot is placed in a plantigrade position in relation to the operating table, parallel to the floor and the detector of the C-arm. This facilitates intraoperative orientation and fluoroscopy without the need to change the position of the standard-sized C-arm for any of the required x-ray planes (dorso-plantar [d.p.]., lateral and oblique) (Fig. 2).

Before sterile washing and draping of the patient’s foot and ankle according to the surgeon’s preference, radiation protection is applied for both the patient and the surgeon. As the surgeon will be seated on the contralateral side parallel to the patient to enable surgery from the medial aspect of the affected foot, a radiation protection mat is attached to the respective side of the operation table. As previously demonstrated in percutaneous coronary procedures, the use of an adjunctive protective shield placed under the operation table is associated with a significant reduction in operator radiation exposure [25]. It does not seem to make any difference what kind of protection is used (curtain vs. drape). We are using a *drape under table* shield that can be easily applied to any standard operating table and does not limit the free movement of the surgeon or those of the C-arm (if needed for additional procedures). Optional radiation protection for the surgeon in addition to a lead apron includes a special x-ray protection thyroid collar, −glasses, and -headpiece [26]. A tourniquet is installed at the thigh but not routinely activated. Activation of the tourniquet is usually not necessary nor recommended as a little blood flow from the osteotomies will help dissipate the heat created by the burr. Nevertheless, a tourniquet might be helpful or required for additional procedures. In the case of the unlikely event of a conversion to open surgical hallux valgus correction a tourniquet might be favourable. Team time-out according to WHO guidelines is carried out with all teams involved during surgery. A pinch test is performed to ensure adequate anesthesia. Visible and palpable anatomic structures, especially prominent subcutaneous veins are marked with a sterile pen. This can help to avoid accidental damage during K-wire and screw placement.

### MIS machine and instruments

The specific equipment required consists of a dedicated high-torque and low-velocity drill that can be fitted with different burr heads. Built-in fluid irrigation is very convenient as there is no need for an extra hand to rinse and cool down the osteotomy. The use of a standard hand-held power tool is not recommended. High *rpm* compensating low torque will lead to heat, necrosis and non-union.

**Fig. 3:**
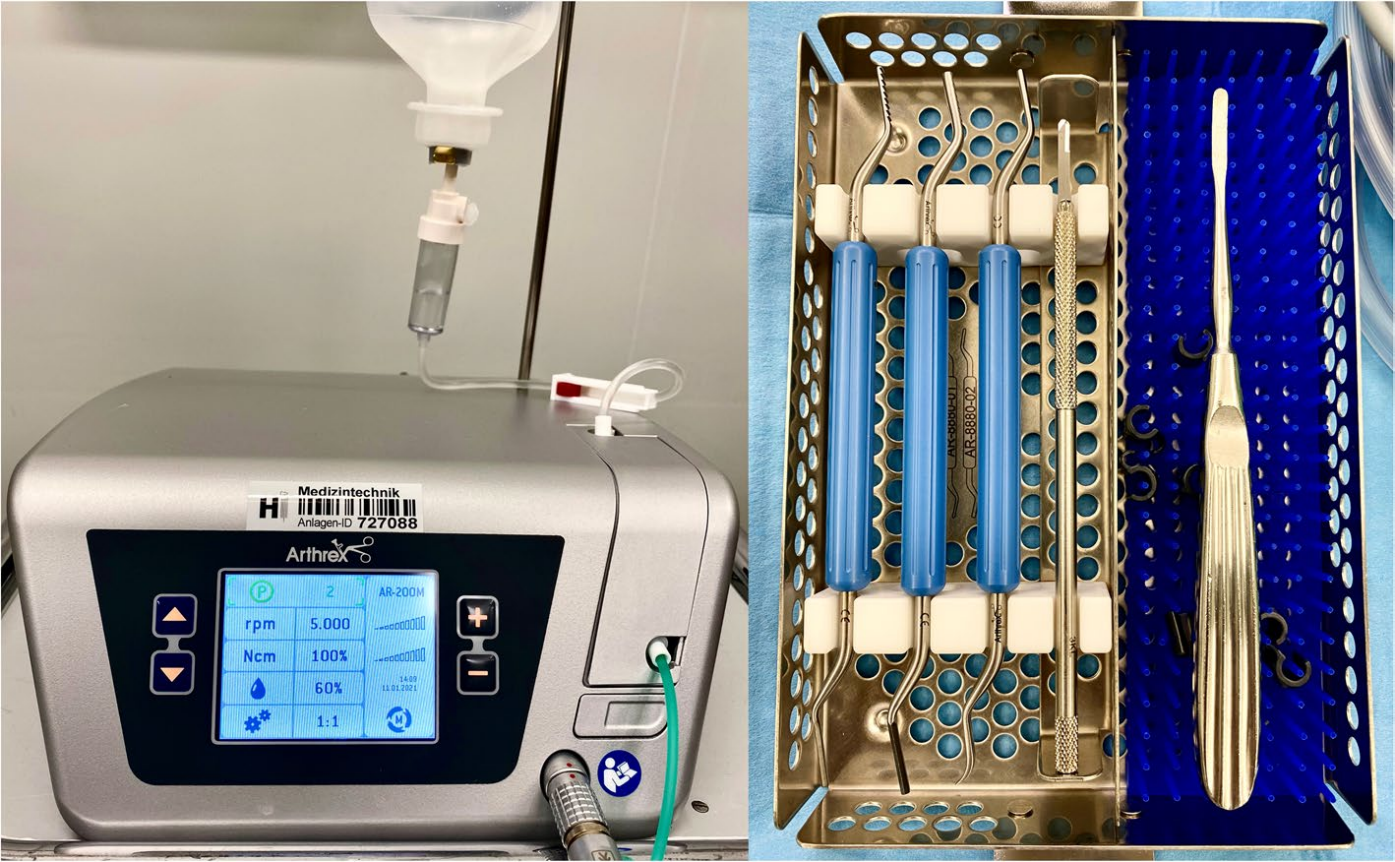
Equipment required for MICA: A MIS console with variable *rpm* and torque, built-in additional irrigation and cooling (Arthrex AR-200 M), a beaver bladed scalpel, different rasps and elevators, cannulated headless screws (not shown)

Other than that, cannulated headless compression screws, preferably bevel-headed, are used for internal fixation. Nowadays several manufacturers are offering specific MICA screws in different diameters. The screws used in our patients are fully threaded, cannulated, and headless compression screws with a diameter of 4.0 mm and 3.5 mm for the Chevron- and 2.5 mm for the Akin osteotomy (*FT Screw*, Arthrex©). Straight and curved elevators facilitate the leverage maneuver and shifting of the metatarsal head. A surgical scalpel with a blade *SM64* is used for the small skin incision and the lateral release (Fig. 3).

### K-wire placement

**Fig. 4:**
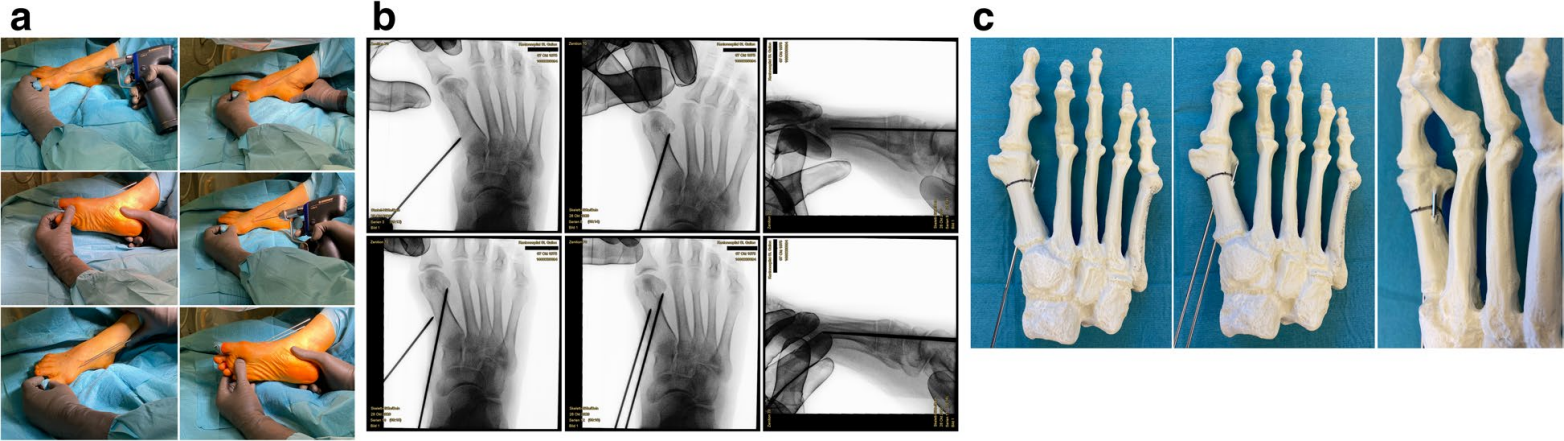
Correct placement of the two 2.0 mm K-wires that prepare the correct placement for the definite guide-wires: **a** Note the prominent subcutaneous veins that are marked with a steriel pen to avoid accidental damage. Intraoperatively, a true d.p. and lateral view can easily be obtained without changing the position of the fluroscope. **b** Fluoroscopic control of the correct K-wire placement. The first/proximal K-wire exits the lateral cortex of MT1 proximal to the osteotomy site. Both K-wires run parallel along the shaft axis of MT1. **c** Sawbone model demonstrating the exit point of the first/proximal 2.0 mm K-wire proximal to the planned osteotomy site (black line)

Surgery commences with the correct placement of the first K-wire introduced percutaneously through the medial base of the first metatarsal. The original 1.1 mm guidewires providing the cannulated screws are usually not strong enough to enable a correct placement and to perforate the lateral cortex at the desired position as they tend to slip and slide in the medullary canal of the metatarsal shaft if entered in a rather low angle in relation to the metatarsal surface. Therefore, a 2.0 mm K-wire or standard 2.0 mm drill bit is used to create the perforations needed for the definite guide-wires which will have to be exchanged later in the procedure (Fig. 4a-c).

The proximal entry point allows for a long anchoring and firm hold of the first screw but requires a very flat course of the wire/screw to exit the lateral cortex just proximal to the planned osteotomy site and to pick up the metatarsal head in its lateral half. The lower the intermetatarsal I/II angle (IMA I/II), the more difficult this step of the procedure might become. This is why mild deformities with a low IMA I/II, compared to moderate to severe *Metatarsus primus varus* conditions, might be more difficult to correct with *the K-wires-first technique* using two parallel screws at the beginning of the learning curve. Bending the K-wire accordingly, which is driven by a standard power tool, and placing the forefoot in an abduction (by pulling on the lesser toes laterally) can help to bring the first K-wire in the correct position. Before penetrating the lateral cortex, fluoroscopic control is necessary in dorso-plantar (d.p.) and lateral view to ensure optimal K-wire placement. Multiple perforations of the lateral cortex will complicate finding the exact spot during the process of replacing the initial K-wires with the guidewires and will unnecessarily weaken the osseous structure. Once the correct position of the K-wire is confirmed fluoroscopically, the lateral cortex is penetrated. A second K-wire is placed accordingly approx. 1.0 to 1.5 cm distally and parallel to the first wire. Perfect placement of the first wire facilitates the safe placement of the second (Fig. 4a-c). As the second wire exits the 1st metatarsal through the osteotomy plane, it will not perforate the lateral cortex.

### Osteotomy

**Fig. 5:**
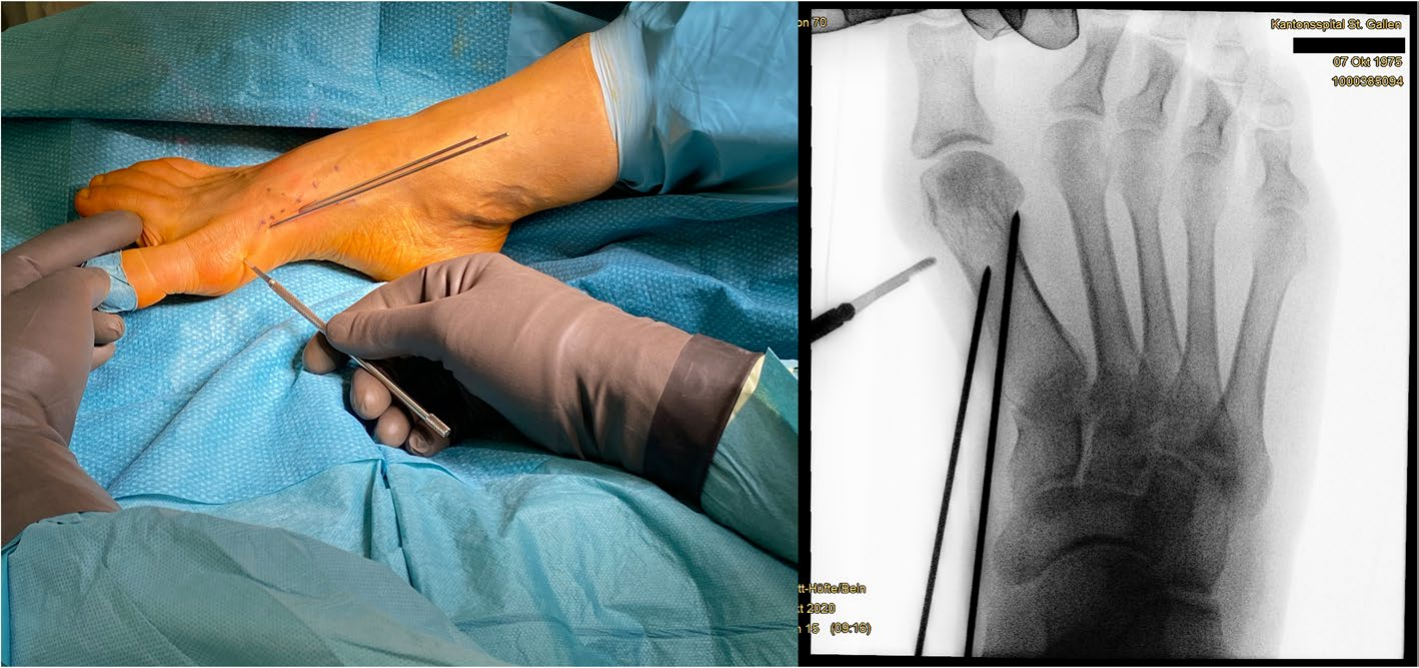
The correct level of the skin incision for the osteotomy portal is checked fluoroscopically

**Fig. 6:**
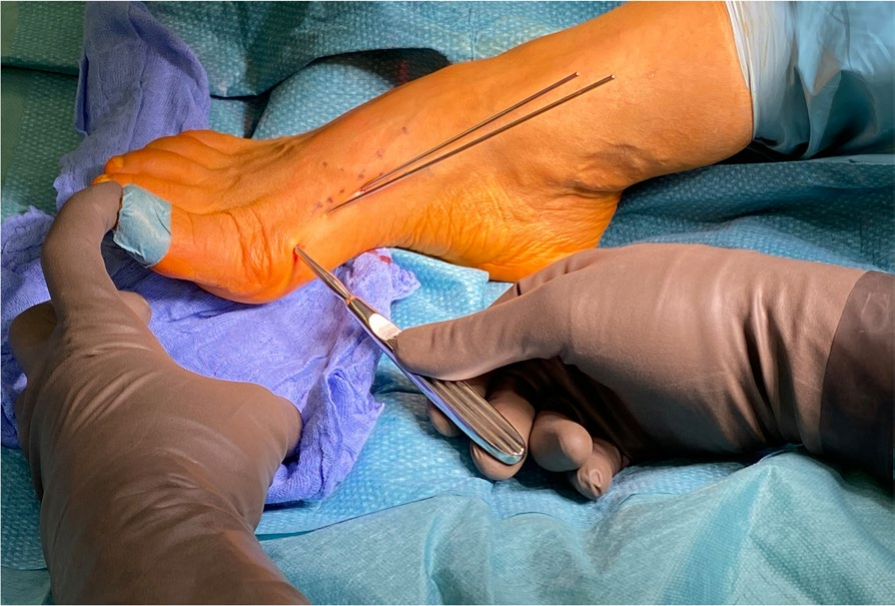
A working space needed to safely work the burr is created by elevating the soft tissues around the osteotomy site with a blunt elevatorium

**Fig. 7:**
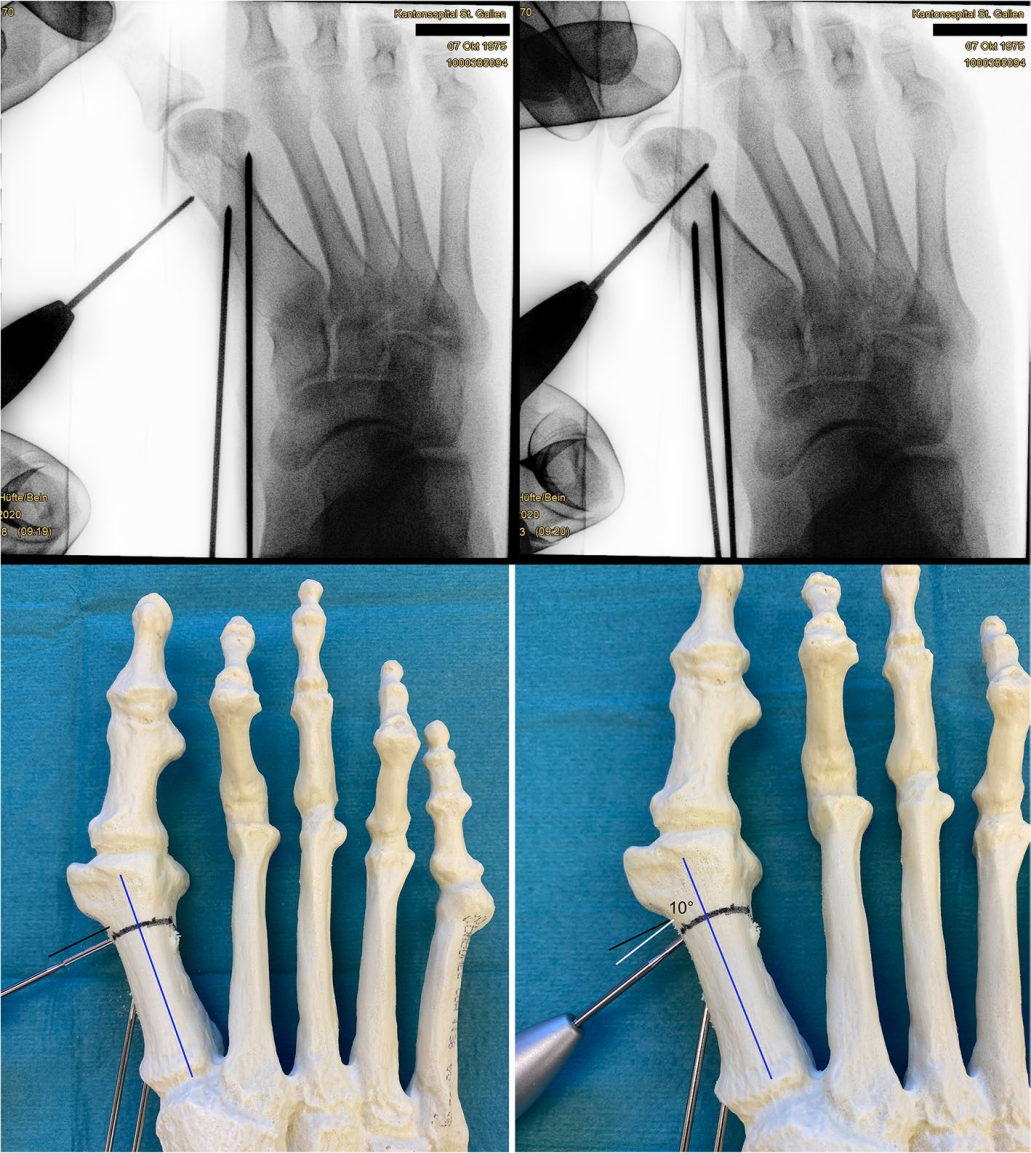
To compensate for the bone loss created by the 2.0 mm ø Shannon burr, the burr is orientated 10° from proximal-medial to distal-lateral (measured by a perpendicular position of the burr in relation to the long axis of MT1)

**Fig. 8:**
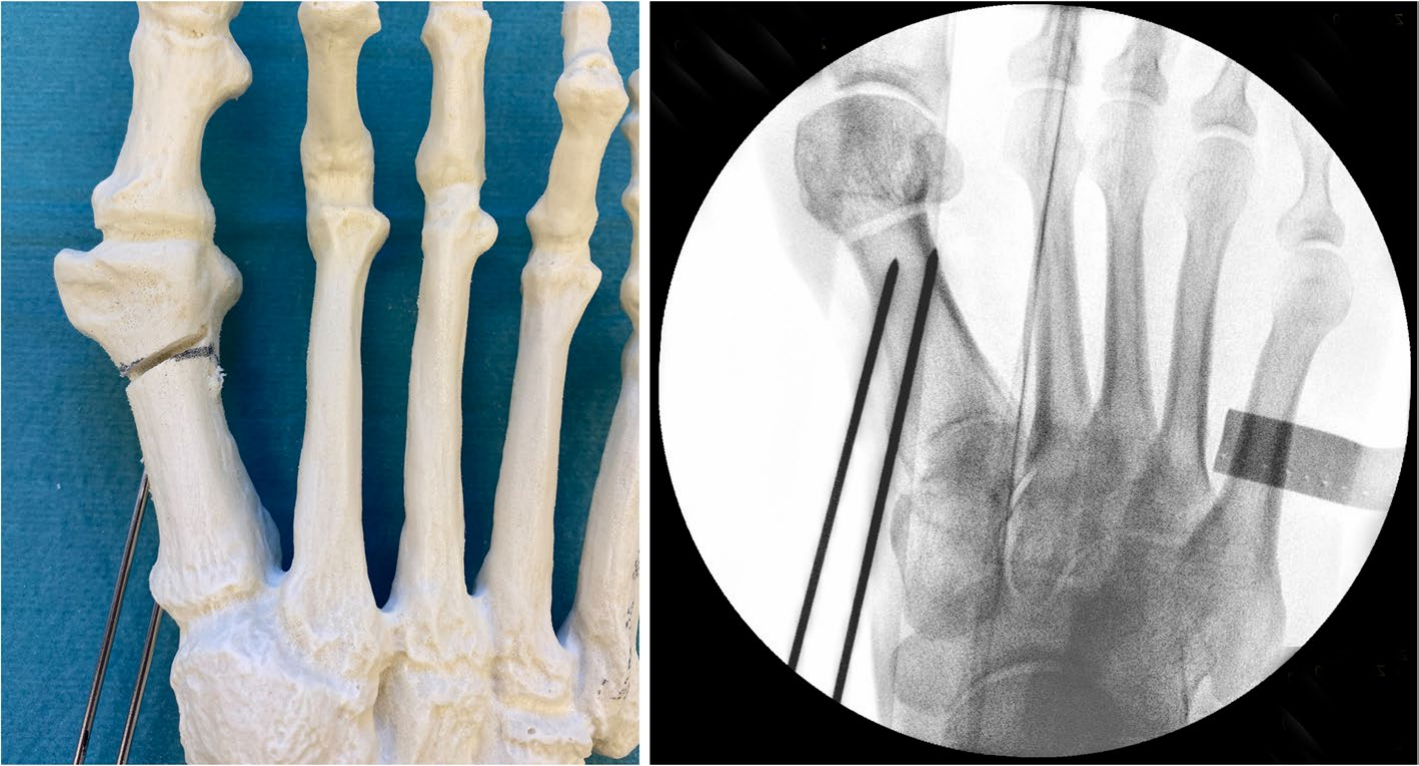
Once the vertical, dorsal cut has been completed, the burr is introduced again to establish the short plantar cut. The saw bone picture demonstrates a dorsal cut where the bone loss created by the burr is compensated by angulation of the osteotomy (left). In the fluoroscopic image the orientation of the osteotomy was deliberately perpendicular to the long axis of MT1 to create a mild shortening in an index +/− situation with mild osteoarthritic changes of MTP1

**Fig. 9:**
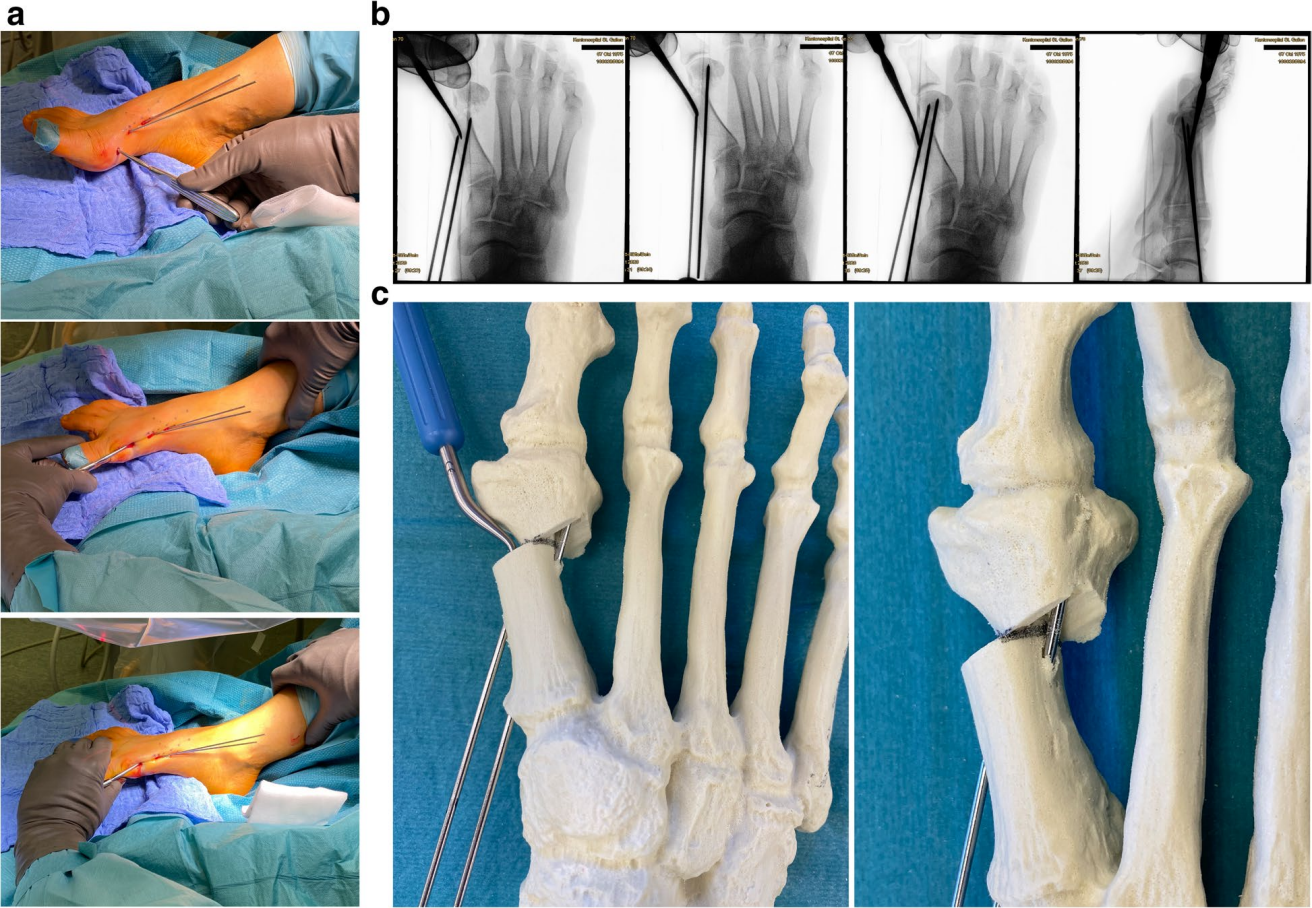
**a** To perform the shifting manoeuvre and lateralisation of the metatarsal head, a small elevatorium is introduced through the portal and placed insides the medullary canal of the MT1 shaft. Care must be taken not to break the osseous hinge in osteoporotic bone. Alternatively, a strong K-wire can be used as a lever. It is important to avoid an unintentional malpositioning in the sagittal plane (dorsalization/excessive plantarization). The metatarsal head can be palpated betwenn the surgeon’s thumb and index finger before the K-wires are advanced. **b** Unintentional malrotation or tiliting of the metatarsal head and worsening of the DMAA or pronation can be counteracted by pulling the big toe into a slight varus and derotating while shifting and advancing the K-wires. **c** Depending on the surgeon’s preferences, a straight elevatorium might be favourable for large amounts of shifting as a curved tool will accomodate the medial prominence of the metatarsal head and decrease the shift

**Fig. 10:**
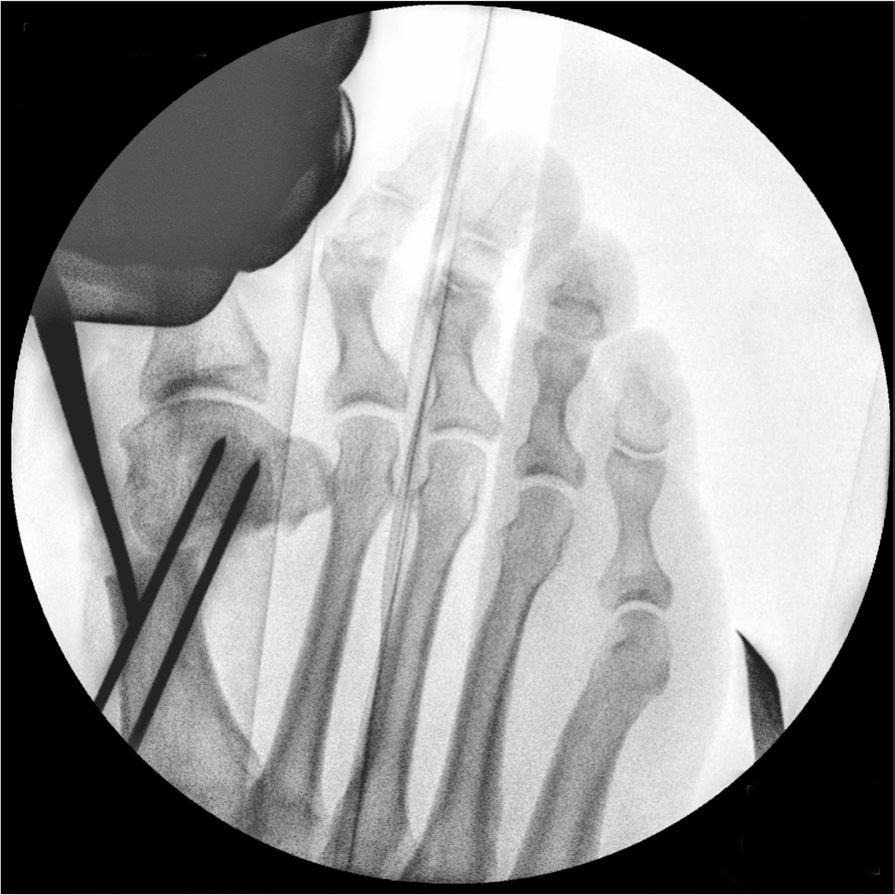
The oblique view, with the leg internally rotated whilst being flexed in the hip and knee and the foot positioned parallel to the operation table, helps to assess the correct K-wire/screw position

The V-shaped Chevron osteotomy of 3rd generation MICA differs from traditional open-surgery Chevron. The dorsal cut is orientated vertically and the plantar cut is rather short. As noted by one of its inaugurators, David Redfern, MICA differs from the classic open Chevron both in position and geometry. The percutaneous biplanar Chevron cut is usually ∼120–130° [16]. A long plantar cut might make the lateral displacement more difficult and, more importantly, can interfere with a safe exit point of the long, proximal screw through the lateral cortex. Recent studies have demonstrated the importance of attention to a correct coronal plane in bunion surgery to achieve sufficient correction and avoid recurrence [27–29]. Biomechanical testing performed by Aiyer et al. demonstrated no significant difference in ultimate load, yield load, and stiffness between transverse and Chevron osteotomy constructs for MIS hallux valgus surgery. This suggests that a uniplanar transverse osteotomy might be favourable to facilitate three-dimensional correction and providing strong fixation at the same time [30]. The site of the osteotomy is also different from open Chevron and is located more proximal, at the neck of the 1st metatarsal and extraarticular. Fluoroscopic control confirms correct placement of the MIS beaver knife used for the medial stab incision (Fig. 5). The portal and corresponding starting point for the biplanar osteotomy is located mid-line of the metatarsal shaft. The soft tissues around the portal for the percutaneous osteotomy are widened with a blunt instrument, e.g. hemostat/mosquito clamp, and the required working space is created with a blunt elevatorium before introducing the tip of the burr to the medial surface of the metatarsal bone. Establishing a working space is mandatory for any percutaneous procedure involving a burr. By circumnavigating the instrument around the neck of the metatarsal bone dorsally and plantarly, the soft tissues adjacent to the bone are freed and elevated allowing safe use of the burr (Fig. 6). To compensate for the bone loss created by the burr (2.0 mm in diameter), 10° of angulation of the burr orientated from proximal-medial to distal-lateral (Fig. 7) is advised if the length of the first ray is to be preserved. Once the burr is introduced and hits the lateral cortex, fluoroscopic control of the desired position of the burr is checked and corrected if necessary. The lateral cortex is penetrated and the dorsal cut is performed in a slow but steady windshield-wiper-movement. Small telescoping movements back and forth help to hear and feel the burr inside the bone. Shortly before the burr is about to exit through the dorsal cortex, the big toe is held in a dorsiflexed position to relax the extensor tendons and neighbouring soft tissues next to the osteotomy. Once the dorsal cut is completed (Fig. 8), the Shannon burr is introduced again through the same portal and the plantar cut is performed, either a short oblique cut or by completing the vertical transverse cut. Sometimes the Shannon burr has to be cleaned of drill dust to ensure unrestricted use and adequate cutting. Analogue to the dorsal cut, shortly before exiting through the plantar cortex of the 1st metatarsal, the big toe is plantarflexed in the MTP1 to relax the flexor tendons. Completeness of the uni- or biplanar osteotomy is checked by moving the metatarsal head and its mobility. The displacement is achieved by introducing an elevatorium into the medullary canal of the metatarsal diaphysis and using the leverage against the medial aspect of the head to push it laterally (Fig. 9a-c). At this point, it is critical to avoid accidental dorsal or plantar malpositioning as well as rotating the head in the transverse plane and unintentionally worsen the DMAA. This can be achieved by both placing the handpiece of the elevatorium in the surgeon’s palm (and maintaining the leverage and displacement), palpating the metatarsal head between the fingertips (feeling its position relative to the long axis of the 1st metatarsal) and, if necessary, counteracting an involuntary rotation or tilting of the metatarsal head by abducting the hallux in a temporary varus position. If necessary, any coronal plane deformity (pronation of the 1st metatarsal) can be addressed at this point. The optimal position is checked fluoroscopically and maintained whilst both K-wires are advanced into the metatarsal head. The correction is controlled again with fluoroscopy in all three planes (Fig. 9b). Especially the oblique view helps to detect plantar protrusion and a suboptimal K-wire placement (Fig. 10) [18].

### Screw insertion

**Fig. 11:**
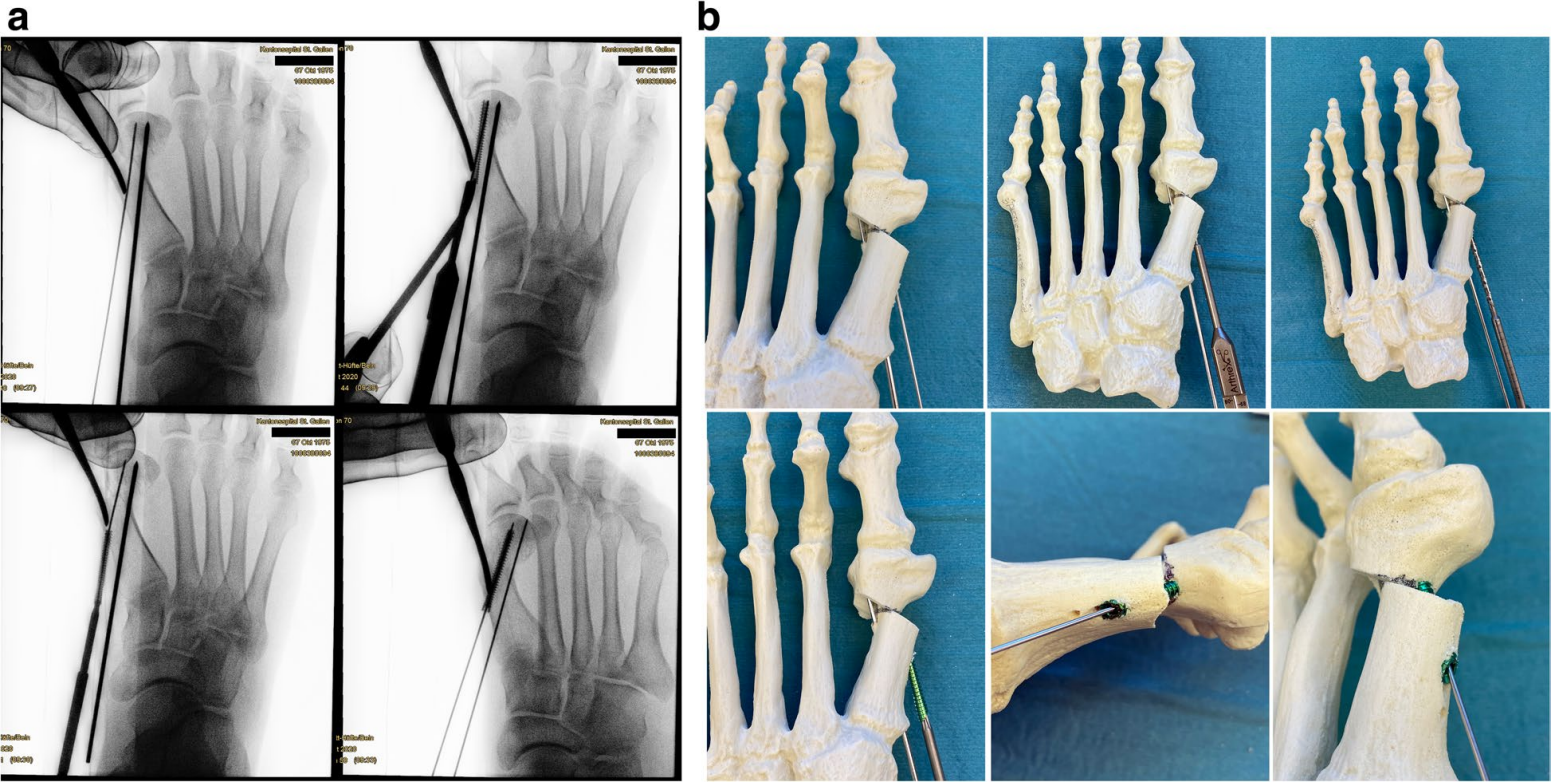
**a** Top left: the 2.0 mm K-wire is exchanged for the guidewire. Top right: Getting the correct length with the corresponding cannulated length gauge and crosschecking by placing the screw on top of the foot. Bottom left: Inserting the first screw and making sure not to bend the guidewire. Bottom right: The oblique view is key to determine the correct length, both proximally (screw head protrusion) as well as distally (intraarticular protrusion). Until the first screw has been safely established, the position of the lever is maintained to avoid secondary dislocation of the metatarsal head. **b** As described above, the corresponding steps to safely place the first screw are demonstrated in a saw bone model

The first 2.0 mm K-wire is replaced by the designated guidewire for the cannulated screw and advanced to the subchondral bone of the metatarsal head. If available, a guidewire with a threaded tip is preferred to avoid dislocation during the drilling process. After extending the skin perforation created by the initial K-wire with the beaver knife to a length of approx. 5 mm, the subcutaneous soft tissue is dissected bluntly and the length of the first screw is determined. Usually, 2-4 mm are subtracted from the measured length to avoid protrusion. Bevel-headed screws, specifically designed for this procedure, can reduce the risk of a later need for hardware removal. For the shorter, more distal screw, overdrilling with the cannulated drill bit is required only for the first cortex. The head remains undrilled to create maximum screw retention in the cancellous bone. For the proximal screw, both cortices must be drilled. Until the first screw has been firmly secured the position may be maintained with the lever as described before. Starting with the distal, shorter screw, medial compression can be achieved, avoiding unintentional deterioration of the DMAA. The position and angulation of the drill bit, relative to the guidewires, should be checked with fluoroscopy to avoid shearing off the wire by the drill (Fig. 11a-b). The oblique view is best suited to detect proximal or distal protrusion of the screws over the level of the metatarsal cortex [18]. Discomfort caused by a protrusion might lead to revision surgery. Testing the passive range of motion of the MTP1 can help to detect restricted movement or crepitations caused by an overlength and plantar/distal perforation of the tip of the screws.

### Removal of the dorso-medial edge of the osteotomy

**Fig. 12:**
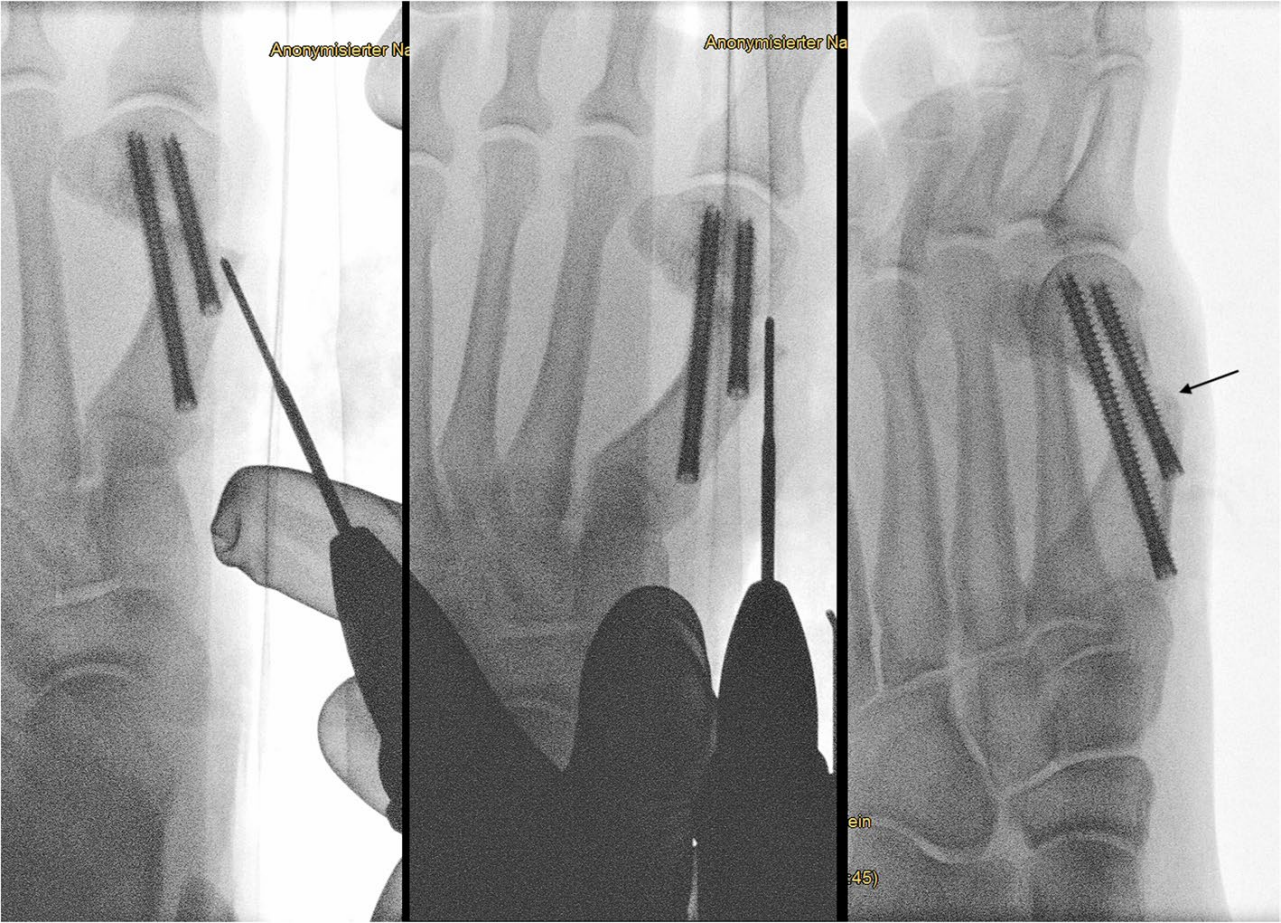
In this case, the edge of the osteotomy is removed with the straight Shannon burr (19.5 × 2.0 mm). Instead of taking it down step by step with the wedge burr, the straight burr performs a small cutting osteotomy parallel to the distal screw. The osseous fragment can be removed with a small hemostat. The oblique view is used to check sufficient removal of the dorso-medial edge (arrow)

**Fig. 13:**
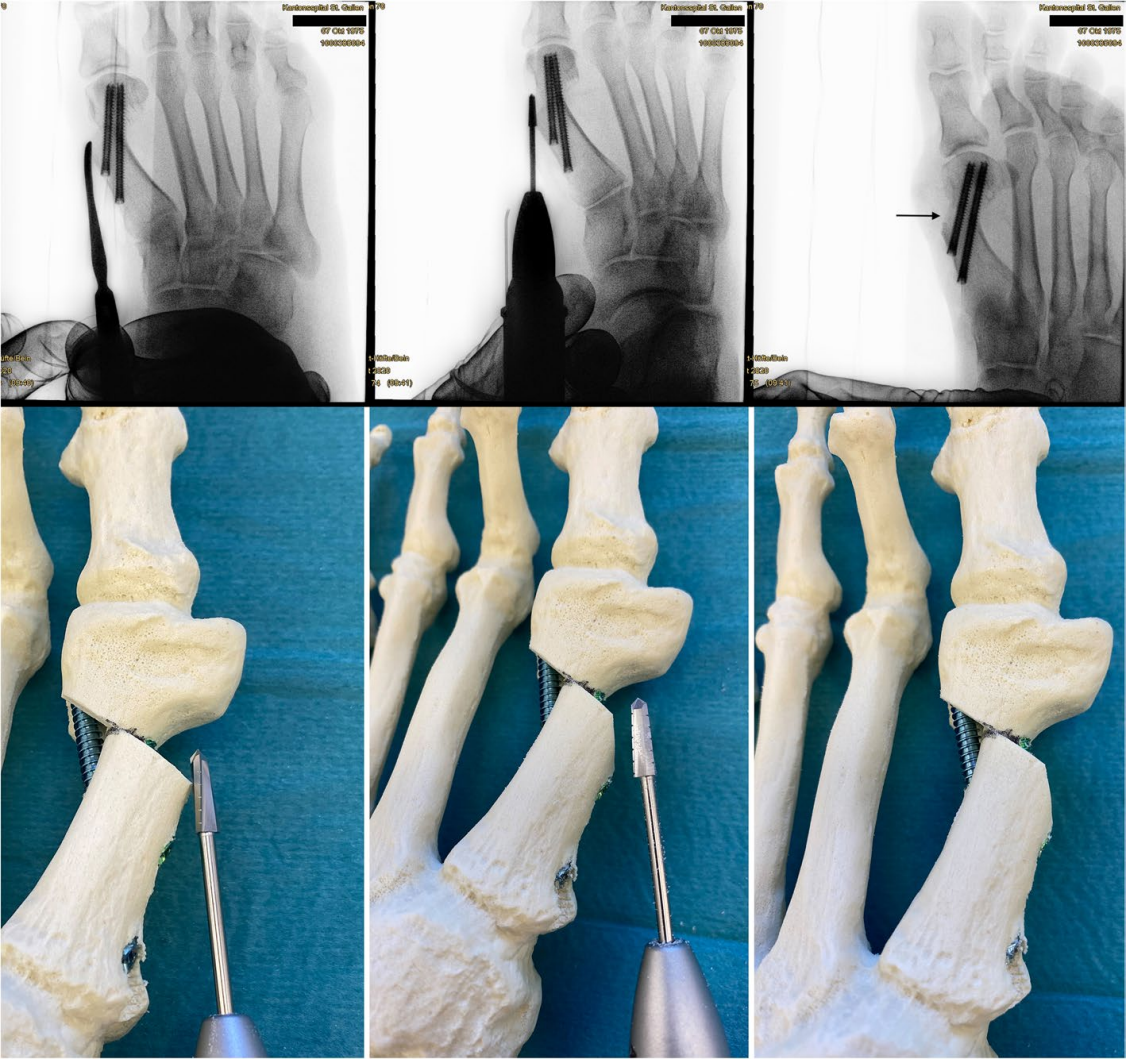
With a straight elevatorium and by blunt dissection, the soft tissues are elevated to create a working space at the dorso-medial edge of the osteotomy. With a 4.3 mm wedge burr, the bony prominence is smoothed down. Afterwards, thorough rinsing is required to wash out bony debris. The oblique view is used to check sufficient removal of the dorso-medial edge (arrow)

As the metatarsal head is routinely displaced further laterally than in open Chevron osteotomy, a medially prominent head / pseudoexostosis is less of a problem than the prominent dorso-medial edge of the osteotomy itself. The medial edge of the osteotomy can be palpated through the skin and should be removed with the use of the burr. Here, two different techniques are possible: Performing a bone cut with the standard 2.0 mm Shannon burr and removing the bony fragment with the help of a mosquito clamp through one of the portals (Fig. 12) or taking down the edge step-by-step with a conical wedge burr (4.3 mm) (Fig. 13). Again, before the use of the burr, a working space has to be created. This can be done by introducing the blunt elevatorium through the second screw portal, almost parallel to the bone. Care must be taken not to weaken the bone stock next to the distal screw excessively with the burr. Furthermore, the wedge burr can easily damage the dorsomedial cutaneous nerve of the hallux and/or prominent subcutaneous veins. Blunt dissection and the creation of adequate working space are mandatory. The muscle belly of the M. abductor hallucis represents a good soft tissue coverage of the plantar half of the medial metatarsal shaft. This is why the reduction is focused on the dorso-medial aspect of the osteotomy. Again, the oblique view is best suited to control the progress and success of this step of the procedure. If deemed necessary, the pseudoexostosis of the metatarsal head can be reduced in a similar way.

### Akin osteotomy

**Fig. 14:**
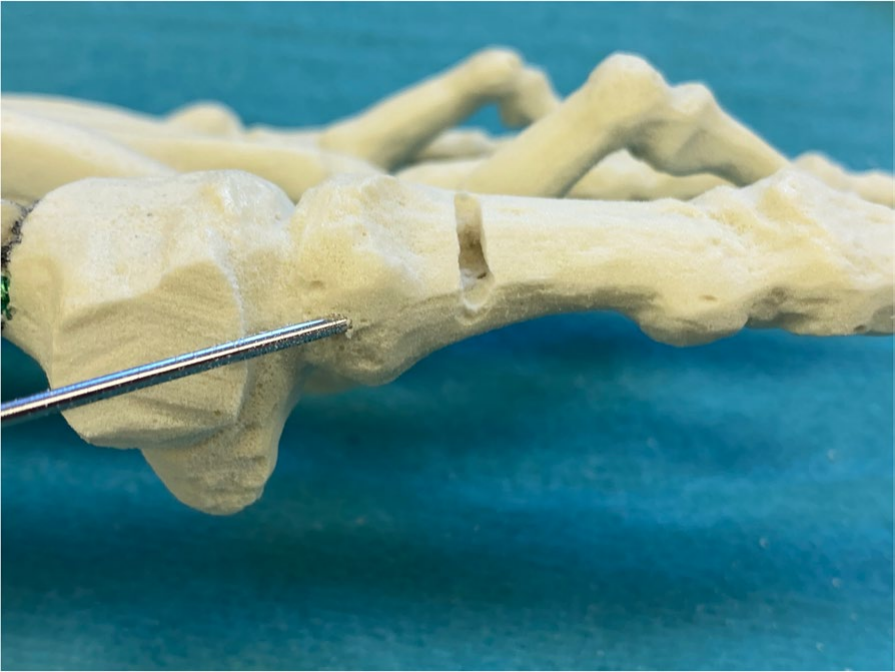
A 1.6 mm K-wire can help to establish the correct entry point for the smaller diameter cannulated compression screw as the original guidewire often is to flexible to penetrate the cortex of P1 at the desired entry point. The osteotomy starts at the medial midline of P1 and the dorsal half

**Fig. 15:**
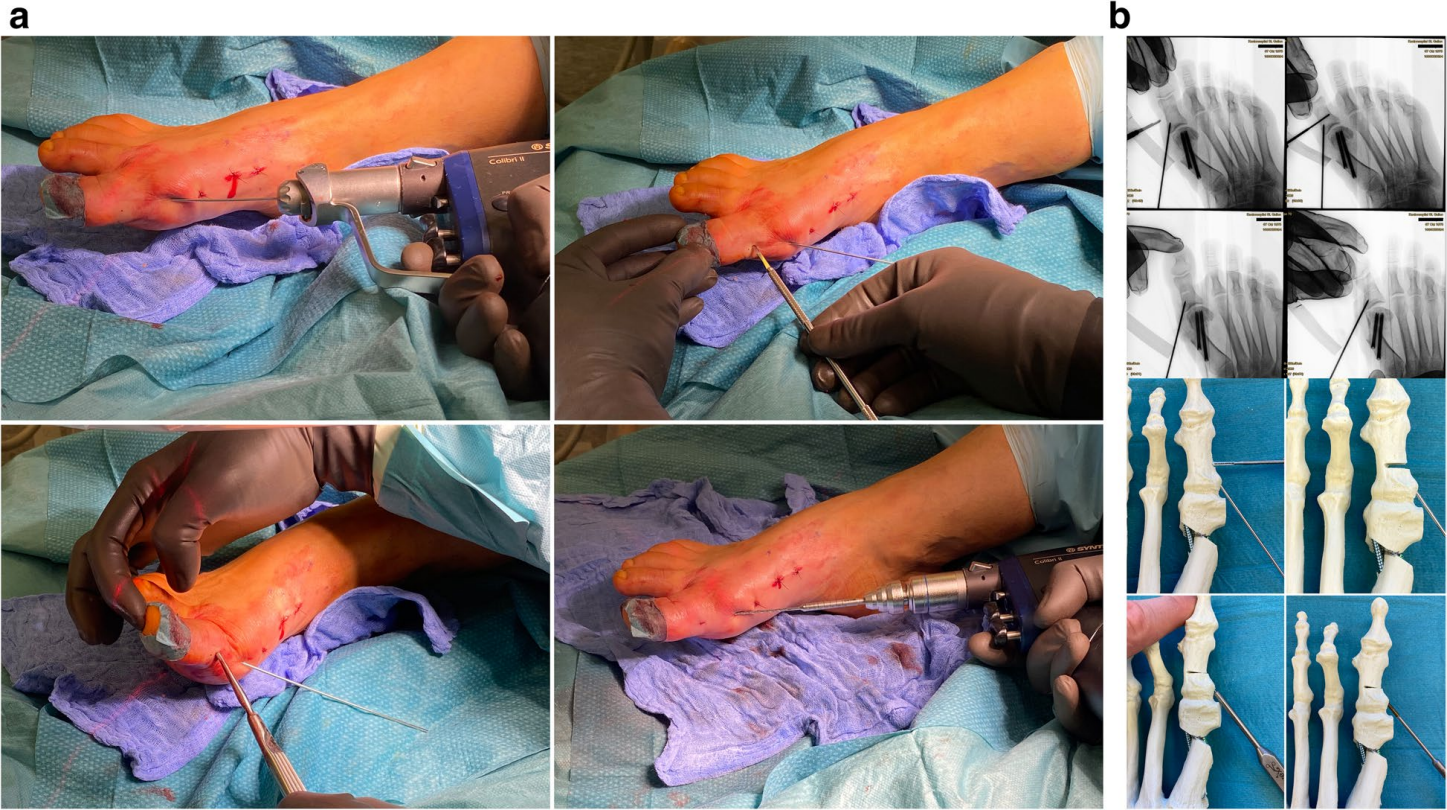
**a** Left to right, top to bottom: 1.6 mm K-wire to establish correct entry point for the guidewire and cannulated screw, fluro-checking the correct level of the osteotomy before the skin incision, creating a working space with a blunt elevatorium, drilling is deemed optional for the soft bone of the base of the proximal phalanx. **b** The osteotomy is proceeded until a medial closing of the osteotomy by passive abduction/varus-stress of the big toe is feasible

The Akin osteotomy represents a medial closing wedge osteotomy of the proximal phalanx (P1) of the great toe and can be performed in an oblique or transverse orientation. The oblique cut, coming from the intersection of the middle to distal third of the medial cortex and aiming to the proximal lateral edge of P1, has the advantage of a (biomechanically) favourable orientation to the screw fixation (approx. 90°), which enters the medial base of P1 and aims to the lateral-distal edge of P1. The osteotomy is longer and requires more time to complete and thus might be more difficult to perform for beginners. The transverse cut is faster and easier regarding a correct orientation. Care must be taken to respect the lateral hinge (cortex) of the medial closing wedge osteotomy. The site of the osteotomy is checked with fluoroscopy and the portal is established with the beaver knife. A working space is created and the 13 × 2.0 mm Shannon burr is introduced. Entering the burr at the midline of the proximal phalanx, the osteotomy starts with the dorsal half (Fig. 14). Same as for the Chevron osteotomy, the adjacent soft tissue structures are relaxed by passive dorsi−/plantarflexion shortly before the burr exits the respective cortex. A cannulated headless screw (e.g. 2.5 mm FT screw, Arthrex©) is used for fixation. A 1.6 mm K-wire can temporarily help to find the correct entry point of the designated guidewire which sometimes tends to slip and slide along the medial cortex of P1 once axial pressure is applied. The correct position of the guidewire is checked and the length of the screw is measured. Overdrilling is deemed optional as the bone of the base of P1 is soft enough for instant screw insertion (Fig. 15a&b).

### Distal soft tissue procedure

**Fig. 16:**
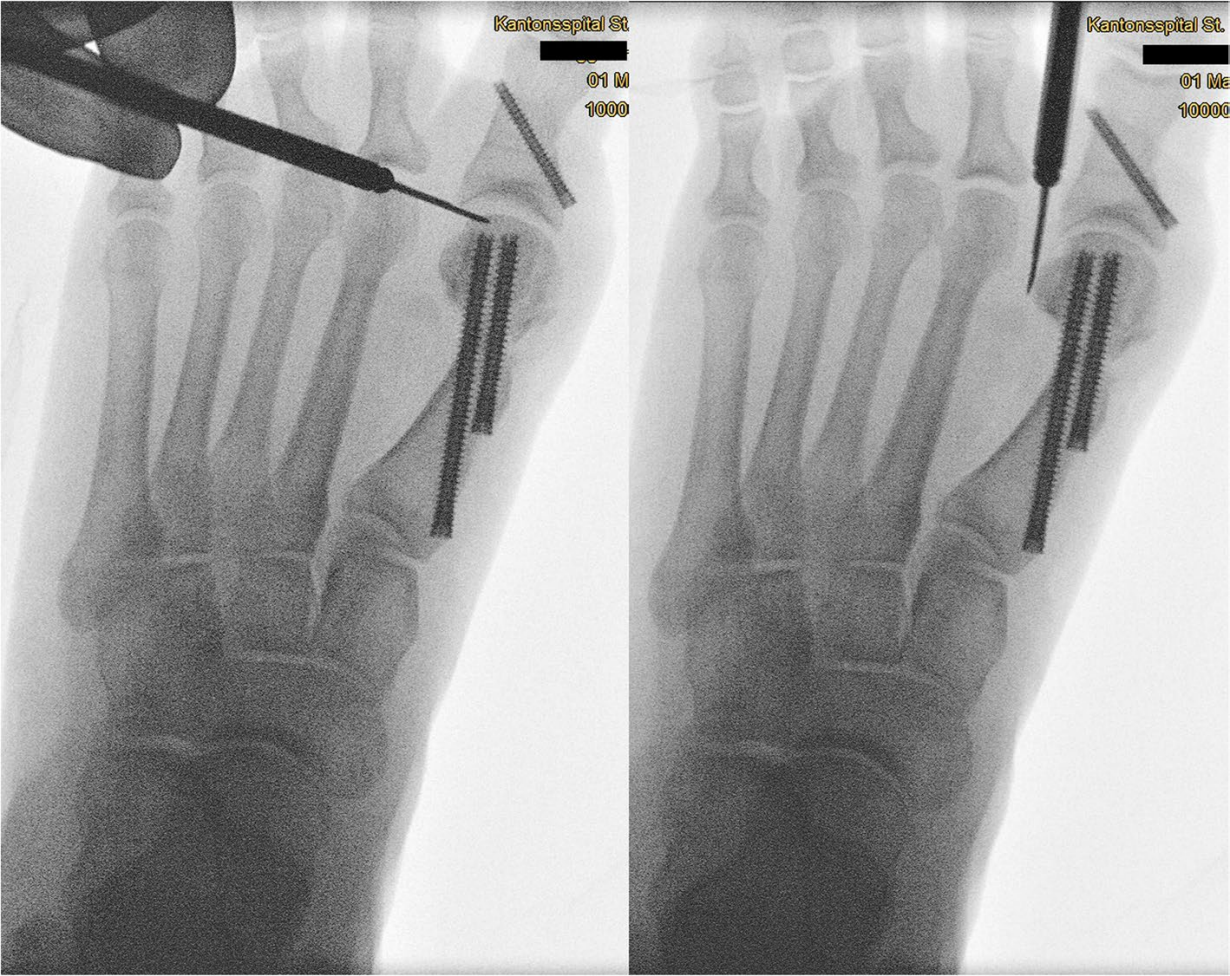
Under fluoroscopic control the level of the joint line is determined and a lateral capsulotomy and release of the the suspension ligament of the lateral sesamoid are performed

If deemed necessary, a distal soft tissue procedure is performed after the osseous correction. According to our experience and other authors [31], a lateral release is less often necessary than in traditional open Chevron osteotomy, where the distal soft tissue procedure represents an integral part of the surgery and is usually performed at first. If the sesamoids are not covered sufficiently by the metatarsal head (don’t forget to check frontal plane rotation earlier [28, 29]), if the great toe still shows a tendency for valgus deviation despite satisfactory osseous correction or if the great toe can not be abducted passively in the MTP1 and feels rather rigid, a distal soft-tissue procedure might be considered and include a lateral capsulotomy and release of the suspension ligament of the lateral sesamoid (Fig. 16). As a final step of the procedure, the insertion of the M. adductor hallucis can last be released percutaneously at the lateral base of P1. As always, this is done under fluoroscopic control.

### Additional procedures performed in combination with MICA

In case of additional percutaneous forefoot procedures like DMMO or MIS lesser toe correction, the surgeon simply changes his position from the side to the end of the operating table. The position of the C-arm remains unchanged. The plantigrade position of the foot, parallel to the operation table, facilitates fluoroscopy, orientation and access to the foot.

### Completion

The portals are thoroughly rinsed with sterile saline solution until all bony debris is removed. If necessary (and lacerated by the screw insertion or use of the burr), the skin around the portals is carefully and sparingly debrided. Skin closure is performed by single-button sutures. At the sites of osteotomies or underlying hardware, we prefer to use sutures over steristrips to achieve a more stable and reliable wound closure.

**Fig. 17:**
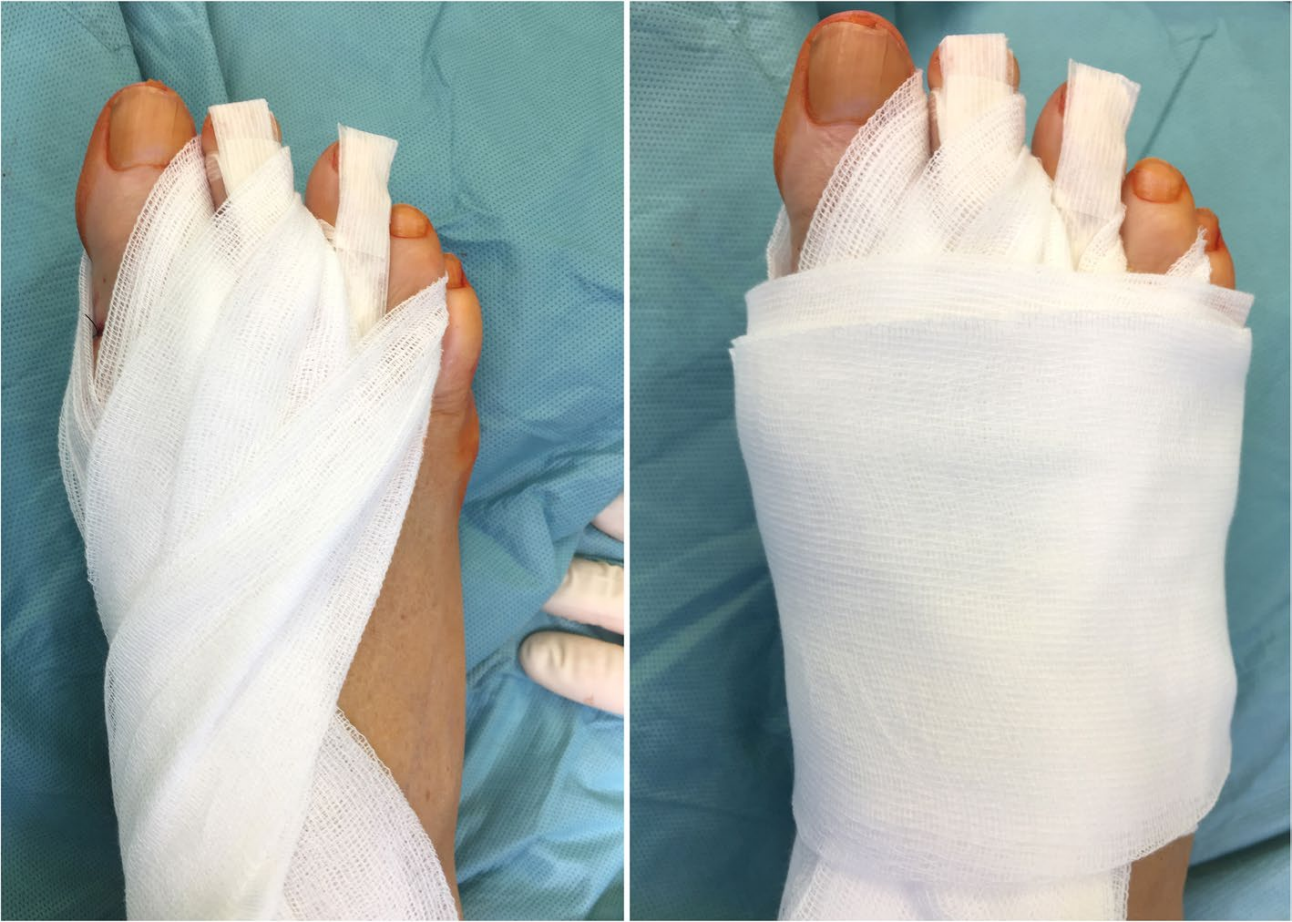
The sterile dressing consists of interdigital compresses that are folded lengthwise and cover the medial skin incisions. In this case, additional percutaneous lesser toe corrections have been performed

A standard sterile forefoot bandage is applied with longitudinally folded 20x10cm gauze compresses placed between the toes. The compresses are applied in a loop form to cover the media surgical wounds. In the first webspace, two compresses are placed to support the alignment of the first ray (Fig. 17). A loose elastic wrap completes the wound care.

### Aftercare

**Fig. 18:**
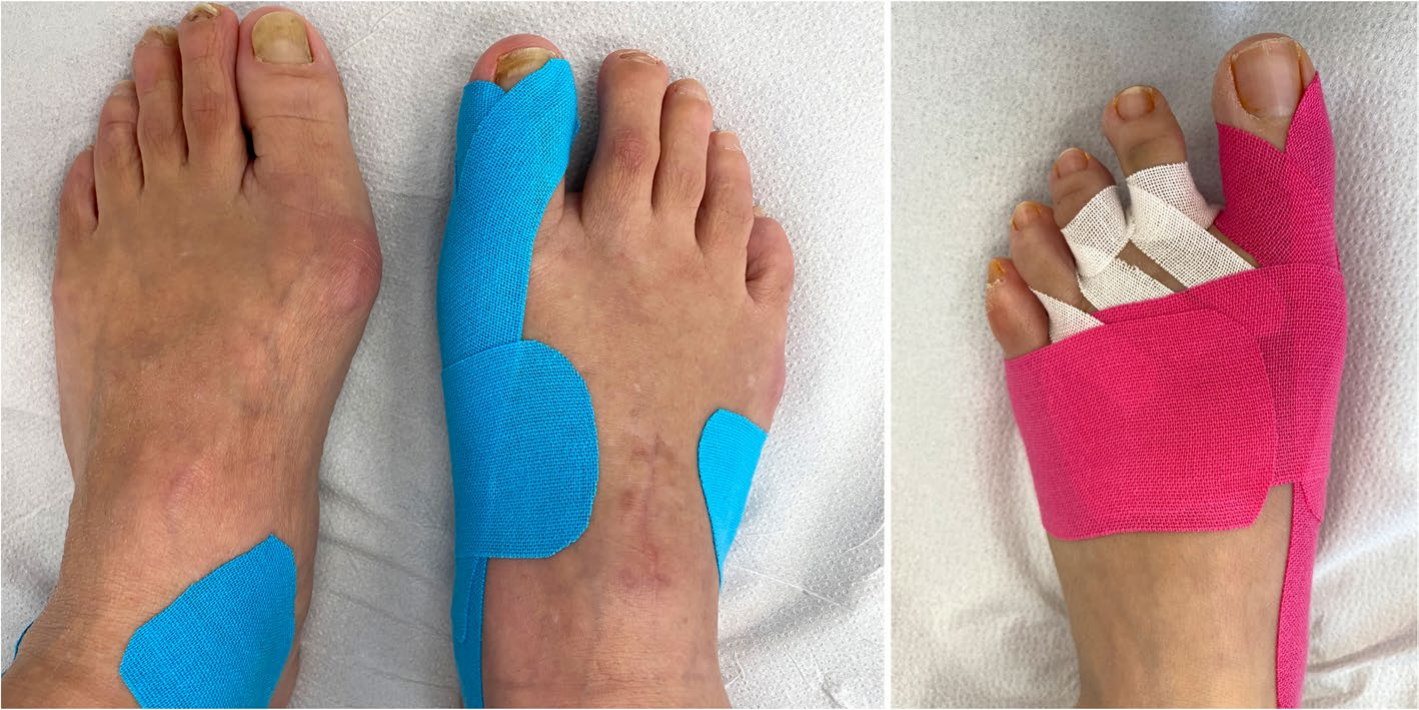
Elastic tapes are applied as soon as the sutures have been removed. To cover the small skin incisions under the tape, small steristrips can be applied. The left picture shows a right foot 6 weeks after MICA. The right pictures demonstrates our choice of taping technique for MICA and additional DMMO 2–4

If possible, we recommend 2 weeks of limited weight-bearing (15 kg) to allow the swelling to subside. An orthotic shoe with a stiff sole is administered for ambulation for 6 weeks. After 2 weeks, the sutures are removed and the dressing is replaced by an elastic tape bandage of the big toe (Fig. 18). X-rays and clinical follow-up are repeated 6 and 12 weeks and 6 and 12 months after surgery. Vitamin C and D are administered generously to support soft-tissue and osseous healing [32]. Vit C supplementation has been associated with improved functional outcomes, decreased post-operative pain, and decreased risk of CRPS development following orthopedic procedures, Vit D is beneficial for wound and bone healing [33, 34].

## Results

All cases were analysed prospectively and will be followed up with clinical and radiological controls for at least 2 years with clinical and radiological after 6 &12 weeks and 6,12 and 48 months respectively. No mal-unions, no malpositioning of the Chevron screws and no metatarsal head necrosis could be observed so far. There was one intraoperative conversion to an open surgical bunion correction in a 71 year-old female patient with osteoporotic bone where the Chevron screws did not find sufficient hold in the soft bone, translating to a 2% conversion rate respectively (1/50).

There were 3 feet in two patients where removal of the Chevron screws was performed after 7, 9, and 12 months due to prominent and disturbing screw heads at the level of the medial cortex. All three osteotomies had fully healed until screw removal. Including the need for screw removal as an indication for secondary revisions, the rate for revision accounted for 6% (3/50). There were no other secondary revision surgeries.

No cases of disturbed wound healing or infection were observed. There were 4 cases of malpositioning of the Akin screws. All four screws showed signs of overlength and plantar/lateral protrusion. None of those cases showed clinical symptoms related to the suboptimal screw placement or required revision surgery.

**Fig. 19:**
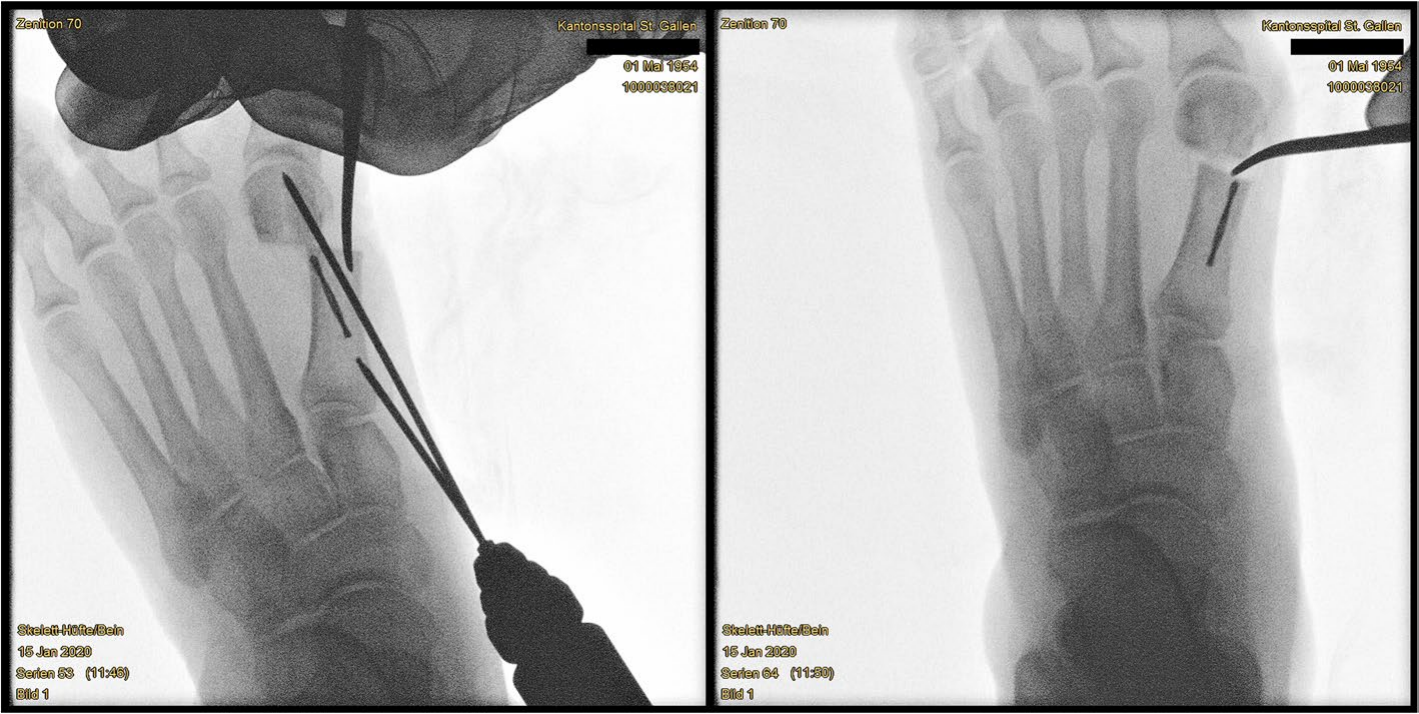
Instead of a 2.0 mm K-wire, a 2.0 mm drill bit can be used to esteablish the correct placement of the guide wires. Its use facilitates penetration of the lateral cortex at the desired position. As it is less flexible than a K-wire, it is more prone to break under stress. In this case the broken drill bit was retrieved via the small portal of the Chevron osteotomy with a hemostat. No conversion to open surgery was necessary

In one case (case 20/50) a 2.0 mm drill bit that was used to prepare the correct entry point for the proximal screw instead of a K-wire broke inside the metatarsal shaft (Fig. 19) but could be rescued percutaneously through the portal of the osteotomy. Ever since, the author used two 2.0 mm K-wires for all other cases.

The preoperative radiological evaluation showed that all cases were classified as moderate or severe deformity, using AOFA’s severity-based radiographic classification of hallux valgus deformity [24]. The mean preoperative IMA was 16.2° (SD 2.7, range 11.0–21.5), the mean HVA 30.6° (SD 5.5, range 21.8–42.1).

Comparing the preoperative and 6-week postoperative radiographs, a significant correction could be achieved. The IMA decreased by a mean of 10.8° (SD 2.8, range 2.4–16.7) from 16.2° to 5.4° (*p* < 0.001)., and HVA by a mean of 22.1° (SD 5.8, range 10.3–36.0) from 30.6° to 8.5°(p < 0.001). Please see Table 3 in the supplementary material section for the radiological outcome of each case.

Radiation exposure to the surgeon was measured using two separate radiation dosimeters exclusively for MICA, one at chest level, the other clipped to the protection glasses. Over more than 3 years, the monthly evaluation did not show any elevated radiation levels.

## Discussion

Although we did not encounter any screw loosening with flat-ended screws as reported by Redfern, we agree with his strong recommendation to use bevel-headed screws [16]. If bevel-headed screws had been used in our series, the revision rate would probably have been lower or nil. We conclude that specifically designed bevel-headed screws that can avoid the protrusion of the screw heads over the medial cortex proximally can lower the risk for revision surgery and thus are strongly recommended.

Compared to other authors, our revision and conversion rate is lower. Chan et al. reported a revision rate of 15.4% (2 feet in 1 patient) requiring early reoperation at 1 month after the initial operation due to mobility at the osteotomy sites. His case series involved only 13 feet in 8 patients and his technique (single screw fixation) differed from the original 3rd generation MICA [35]. Holme et al. reported a complication rate of 10%, with 4 patients requiring Akin screw removal after bony union due to soft tissue irritation [36]. Jowett et al. documented 22 complications in 106 patients requiring surgery, most notably due to screw malpositioning/protrusion, and an overall reoperation rate of 15% [18]. Altenberger et al. provided a detailed description of MICA in the German literature in 2018. Their complication rate in 43 cases was reported to be equal to the conventional open surgery technique, yet missing to present concrete numbers [37]. Frigg et al. reported a reoperation rate of 27% (13/48) in their MICA group, mostly associated with screw removal [10]. Similar to Chan and other authors [35, 38], their percutaneous bunion repair differed from the original 3rd generation MICA technique, complicating direct comparison. The most recent publication of 3rd generation MICA by Lewis et al. reports an overall complication rate of 21.3% in 292 cases and an “all-cause screw-removal rate” of 6.3% [17]. Comparison to other generations or techniques of minimally-invasive hallux valgus repair seems difficult as indications and aftercare can differ significantly. Yet, as Biz et al. demonstrated in their recent publication, not only 3rd generation MICA seems capable of adequate correction of moderate to severe hallux valgus deformity with a minimally-invasive technique. Their long-time follow-up of the MIIND technique (Minimally Invasive Intramedullary Nail Device) also showed favourable results with an implant removal rate of 6% (6/100) and a high potential for correction (mean IMA correction 9.9°, mean HVA correction 27.1°) [39]. Regarding the amount of correction of IMA and HVA, our series demonstrated a high potential for correction, even for severe hallux valgus deformities: The IMA decreased by a mean of 10.8°from 16.2° to 5.4° and HVA by a mean of 22.1° from 30.6° to 8.5°. Comparing those values to the existing literature of MICA, Vernois et al. reported a mean correction of the IMA of 9.0° (from 14.5° to 5.5°) and the HVA of 26.4° (33.7° to 7.3°) [6], Chan et al. reported a mean correction of the IMA of 3.7° (from 13.9° to 10.2°) and HVA of 19.5° (from 30.4° to 10.9°) with their technique [35], Holme et al. reported a mean decrease of the IMA of 6.5° (from 13.2° to 6.7°) and the HVA of 19.6° (from 31.7° to 12.1°) [36] and Frigg reported a mean correction of the IMA of 7° (from 13° to 6°) and of the HVA of 18° (25° to 7°) [10]. It is interesting to note, that modifications of the 3rd generation MICA technique, that only use one screw (Chan et al., Frigg et al. [10, 35]) tend to produce less correction compared to those who perform the original two-screw-fixation for the chevron osteotomy. This can be explained by the fact that a two-screw technique provides a stronger fixation and thus allows a greater displacement of the head [6].

### Limitations

There is a limited number of studies on minimally-invasive hallux valgus surgery. Moreover, 3rd generation MICA has seen various modifications, often deviating from the original report, complicating comparison. Although 50 cases represent a limited number of patients, our number of cases is comparable [10, 31, 40] or superior [35, 36] to most previous reports. Due to the global pandemic and its impact on daily clinical practice, the indications for a highly elective procedure such as MICA were low. As the focus was set on a detailed description of the surgical technique and its immediate complications, no functional outcome analysis has been discussed in this article. For the future, high-level evidence studies with a longer follow-up are needed to shed more light on a promising technique that will bring real benefits to the affected patient.

## Conclusion

The learning curve of 3rd generation MICA has been reported to be prolonged and the technique is generally considered an advanced and demanding procedure [18, 40]. This report aims to thoroughly illustrate each surgical step and provide a detailed description of a modification of MICA. We conclude that the *K-wires-first technique* can help to reduce hardware malposition of the Chevron screws and facilitates handling with a standard-sized C-arm. As demonstrated before by other authors, there is a high potential for correction even for severe deformities. Further efforts will go into describing our learning curve as well as the mid- to long-term radiological and functional outcome of this modification of 3rd generation percutaneous hallux valgus correction.

## Supplementary Information

**Additional file 1: Table 1.** Pearls and pifalls of the “K-wires-first” modification of 3rd generation MICA.

**Additional file 2: Table 2.** Advantages and disadvantages of the “K-wires-first” modification of 3rd generation MICA.

**Additional file 3: Table 3.** Demographics and radiological outcome of all 50 cases. To protect anonymity, the individual patient’s age was changed to age-ranges.

## Data Availability

The datasets generated and/or analysed during the current study are not publicly available due to patient personal health confidentiality agreement but are available from the corresponding author on reasonable request.

## Abbrevations

DMMO: Distal Minimally-invasive Metatarsal Osteotomy
d.p.: dorso-plantar
HVA: Hallux valgus Angle
IMA: Intermetatarsal Angle (I/II)
MICA: Minimally-invasive Chevron & Akin
MICO: Minimally-invasive Calcaneal Osteotomy
MIS: Minimally-invasive Surgery
MTP1: 1st Metatarso Phalangeal Joint
SD: Standard Deviation
TMT1: 1st Tarso Metatarsal Joint
WHO: World Health Organisation
