# Supplementary material for: 3rd generation MICA with the “K-wires-first technique” - a step-by-step instruction and preliminary results
Source: BMC Musculoskelet Disord. 2022 Jan 18;23:66. doi: 10.1186/s12891-021-04972-5 (PMC8767719; doi:10.1186/s12891-021-04972-5)
Supplement: Supplementary file 1 — Additional file 1: Table 1. Pearls and pifalls of the “K-wires-first” modification of 3rd generation MICA. [file 12891_2021_4972_MOESM1_ESM.docx]

*Tab. 1: Pearls & Pitfalls*

- The Shannon burr is a single-use device and not intended for re-sterilization. Using the burr multiple times can lead to breakage or, more likely, non-union of the osteotomy. A blunt burr will create heat, heat will create necrosis and necrosis leads to non-union.
- The position of the burr is not only controlled fluoroscopically but also tactically and acoustically. Both the haptics and the sound of the burr differ from osseous or soft-tissue environment.
- Dorsalization of the metatarsal head must be avoided. Shortening of the first ray can be prevented by adapting the orientation of the osteotomy. In selected cases, as in *index-plus* situations [41], mild-to-moderate intraarticular degenerative changes or a severe bowstring phenomenon of the extrinsic flexor and extensor tendons, a mild shortening created by the burr´s diameter might be considered.
- In osteoporotic bone a brisk leverage manoeuvre with an elevatorium inside the metatarsal shaft may result in avulsion and damage to the medial cortex at the level of the osteotomy. Alternatively, a more flexible 2.0mm K-wire can be used instead.
- Depending on the amount of displacement, smoothening down of the edge of the osteotomy might be more important than a bunionectomy. Here, trimming down the dorsomedial edge of the osteotomy with a conical wedge or straight Shannon burr must be performed carefully not to compromise the hold of the screw in the cortex.
- The intraoperative oblique x-ray view is best suited to detect proximal and distal screw protrusion and helps to reduce implant-related complications [17]. It is also the best way to see the extent of removal of the dorso-medial bone edge at the level of the osteotomy.
- Internal fixation of the Akin osteotomy with a cannulated headless compression screw is strongly recommended. As stated by Redfern, without fixation, there is a tendency for the Akin osteotomy to displace dorsally in the postoperative period which can lead to a dorsiflexion malunion [26].
- A lateral release is optional and performed, if necessary, after completion of the osseous correction.
