# Supplementary material for: 3rd generation MICA with the “K-wires-first technique” - a step-by-step instruction and preliminary results
Source: BMC Musculoskelet Disord. 2022 Jan 18;23:66. doi: 10.1186/s12891-021-04972-5 (PMC8767719; doi:10.1186/s12891-021-04972-5)
Supplement: Supplementary file 2 — Additional file 2: Table 2. Advantages and disadvantages of the “K-wires-first” modification of 3rd generation MICA. [file 12891_2021_4972_MOESM2_ESM.docx]

*Tab. 2: Advantages/ Disadvantages*

- With the *K-wires-first technique*, the osteotomy is only performed once the position of the guidewires is perfect. Until then conversion to open bunion correction is possible without compromises. Overall, this could be beneficial for novices with 3rd gen. MICA and may help to improve the quality of patient care.
- Placing the foot at the same level as the operation table in combination with the use of standard-sized C-arm greatly facilitates handling. All three radiographic planes (d.p., oblique and lateral) can easily be obtained without moving the C-arm which helps to diminish operation time and eliminates the need for an assistant operating the X-ray machine. A mini C-arm might be favourable concerning radiation exposure but not all surgeons have access to a mini C-arm. Moreover, with adequate radiation protection, no elevated radiation levels could be detected in our series.
- If accompanying open procedures (e.g. TMT2 fusion) requiring a straight leg in supine position are performed, the support under the knee can be removed without repeating the sterile washing and draping process.
- The *K-wires first* technique requires more surgical steps compared to the original procedure [6,7]. More surgical steps might translate to a longer operation time and more radiation. An analysis of the author´s learning curve in regard to operation time and amount of x-ray controls will follow.
