# Supplementary material for: 3rd generation MICA with the “K-wires-first technique” - a step-by-step instruction and preliminary results
Source: BMC Musculoskelet Disord. 2022 Jan 18;23:66. doi: 10.1186/s12891-021-04972-5 (PMC8767719; doi:10.1186/s12891-021-04972-5)
Supplement: Supplementary file 3 — Additional file 3: Table 3. Demographics and radiological outcome of all 50 cases. To protect anonymity, the individual patient’s age was changed to age-ranges. [file 12891_2021_4972_MOESM3_ESM.pdf]

Patient 1-50	Age range	Localisation	Sex	IMA preop	IMA 6 weeks postop	HVA preop	HVA 6 weeks postop
1	65-75	right	f	16,1	5,5	30,1	8,7
2	45-55	left	f	17,4	5,6	29,3	6,9
3	55-65	right	f	21,4	7,1	40,6	4,6
4	75-85	left	f	17,6	4,9	34,7	7,1
5	35-45	right	f	21,4	6,1	32,3	4,4
6	55-65	left	f	19,1	8,8	29,2	4,5
7	55-65	left	f	15	5,4	30	4,4
8	55-65	right	m	15,7	7,4	28,8	9,6
9	55-65	left	f	12,5	4,3	39,2	10,2
10	35-45	left	f	16	8,2	21,8	7,4
11	55-65	right	f	14,5	4,8	30	11,8
12	65-75	right	f	15,3	2,9	30,8	8,6
13	55-65	left	f	9,7	7,3	31	14,2
14	75-85	right	m	14,1	3,1	26,1	6,1
15	65-75	right	f	19,2	6,9	37,6	16,3
16	65-75	left	f	18,9	6,8	42,1	8,9
17	35-45	left	f	19,7	5	31,4	13,5
18	55-65	right	f	13,9	4,9	23,6	13,3
19	55-65	left	f	17,6	2,8	36,1	6,7
20	35-45	right	f	14,8	8,3	24,2	8,6
21	55-65	left	f	17,5	8,2	23,5	9,6
22	25-35	left	f	15,8	5,7	24	3,3
23	65-75	left	f	12	4	25,3	11,7
24	45-55	right	f	14	3,9	30	8
25	65-75	right	f	21,5	4,8	40,8	7,3
26	65-75	right	m	16,2	5,6	29,7	7,9
27	55-65	right	f	18,1	8,2	34,8	4,8
28	55-65	right	f	13,8	3,7	29,3	9,8
29	55-65	left	f	15,5	7,9	35,6	14,3
30	65-75	right	m	15,3	6,1	25,4	5,3
31	55-65	left	f	14,4	2,8	24,4	3
32	18-25	left	f	14,5	3,6	29,3	7,8
33	65-75	right	f	20,5	5,4	34,8	2,6
34	18-25	right	f	13,9	4,6	25,7	6,7
35	55-65	left	m	13,5	1,5	28,6	12,9
36	75-85	right	f	18,8	7,7	35,9	9,4
37	35-45	right	f	17,5	5,7	25,9	4,7
38	55-65	left	f	15,6	7,5	23,7	4,2
39	45-55	left	f	18,5	2,3	30,7	11,1
40	45-55	left	m	17	8,5	35,2	11,3
41	45-55	right	f	18,1	2,2	37,5	1,5
42	55-65	right	f	18,2	2,5	40,7	15,7
43	55-65	left	f	19	6,1	34	10,9
44	25-35	right	f	14,5	3	22,8	2,7
45	55-65	left	m	10,5	2,8	32,2	12,6
46	45-55	right	f	14,4	5,6	23,6	6,4
47	45-55	left	f	14,8	5,2	25,4	8,1
48	55-65	left	f	17,4	8,4	34,2	15
49	45-55	right	f	17,9	5,3	35	12,9
50	45-55	right	f	13,8	4,3	23,7	5,4
